# Evaluation of EuroSCORE II to Determine the Prognosis of Patients With Moderate-To-Severe Aortic Stenosis: A Long-Term Retrospective Study

**DOI:** 10.31083/RCM42757

**Published:** 2026-02-04

**Authors:** Xianzhen Cai, Danchun Hu, Baoxin Yan, Jinhao Chen, Weiwen Li, Ying Lin, Run Lin, Junjun Ye, Jiating Su, Shuangshuang Tong, Xiaojun Huang, Bin Xie, Xiaodong Zhuang, Jianying Chen, Jilin Li

**Affiliations:** ^1^Department of Cardiology, Second Affiliated Hospital of Shantou University Medical College, 515000 Shantou, Guangdong, China; ^2^Shantou University Medical College, 515041 Shantou, Guangdong, China; ^3^Center for Cardiovascular Diseases, Meizhou People's Hospital, Meizhou Academy of Medical Sciences, 514031 Meizhou, Guangdong, China; ^4^Department of Cardiology, The First Affiliated Hospital of Sun Yat-Sen University, 510080 Guangzhou, Guangdong, China; ^5^Department of Cardiology, Affiliated Hospital of Guangdong Medical University, 524001 Zhanjiang, Guangdong, China

**Keywords:** aortic valve stenosis, EuroSCORE II, all-cause mortality, aortic valve replacement

## Abstract

**Background:**

Aortic stenosis (AS) is a prevalent heart valve disease; however, morbidity and mortality are significantly reduced by aortic valve replacement (AVR). The European System for Cardiac Operative Risk Evaluation II (EuroSCORE II) is used to assess perioperative mortality risk in patients with severe AS undergoing AVR. This study aimed to evaluate the prognostic value of EuroSCORE II for long-term all-cause mortality in Chinese patients with moderate-to-severe AS, determine whether AVR affects this prognostic value, and identify the best cut-off value for low-risk EuroSCORE II patients without AVR.

**Methods:**

A total of 544 patients with moderate-to-severe AS were divided into four groups based on the associated EuroSCORE II value (cut-off of 4%) and whether the patient had previously undergone AVR. Kaplan–Meier survival analysis, Cox regression, and subgroup analyses were performed to assess the association between EuroSCORE II and all-cause mortality. A receiver operating characteristic (ROC) analysis was used to determine the optimal cut-off value for predicting mortality.

**Results:**

A total of 132 (24.3%) participants reached the endpoint during a median follow-up of 3.45 years. Patients with a EuroSCORE II ≥4% who did not undergo AVR had significantly higher all-cause mortality rates compared to other groups (55.4% vs. 6.5%, 32.7%, and 13.4%;* p < *0.001). Kaplan–Meier analysis confirmed these findings (log-rank test, *p* < 0.001). Cox regression showed a 6.89-fold increased risk in patients without AVR and higher EuroSCORE II values (hazard ratio (HR), 6.891; 95% confidence interval (CI), 3.083–15.401;* p < *0.001). The optimal cut-off value for predicting mortality in patients without AVR was 2.23% (area under the curve (AUC), 0.675).

**Conclusions:**

Both EuroSCORE II (cut-off value of 4%) and AVR status were independently associated with the long-term prognosis of patients with moderate-to-severe AS.

**Clinical Trial Registration:**

NCT06069232, https://clinicaltrials.gov/study/NCT06069232.

## 1. Introduction

Aortic stenosis (AS) is the most prevalent heart valve disease, causing significant morbidity and mortality in the elderly due to aging of the population [1, 2, 3]. Its prevalence exceeds 10% in individuals aged over 65 in US and European populations [4, 5, 6], although the prevalence in China may be lower, according to the results of a single-centre retrospective study of the echocardiographic data of 287,556 patients [7]. The initial stages of the disease are characterized by the progression of valvular lesions, involving endothelial cell damage, infiltration with lipids and macrophages, lipid oxidation, and subsequent fibrosis and calcification, which ultimately leads to obstruction of the aortic valve [6, 8].

To date, due to the lack of specific medications for treating or preventing the progression of AS, aortic valve replacement (AVR) which includes surgical aortic valve replacement (SAVR) and transcatheter aortic valve implantation (TAVI) is recommended for patients with severe symptomatic disease [9, 10].

EuroSCORE II is a logistic risk model developed in 2011 from 22,381 patients in 22 European centers and weights 18 peri-operative variables (age, renal function, cardiac status, etc.) [11]. It was originally developed for perioperative risk stratification in cardiac surgery [11, 12], but had also been validated for predicting in-hospital mortality and short-to-medium-term mortality after AVR [13], coronary artery bypass grafting (CABG) [14]. However, there are limited studies assessing the long-term prognostic value of EuroSCORE II for patients with moderate-to-severe AS, regardless of whether they undergo AVR (SAVR or transcatheter aortic valve replacement (TAVR)), especially in Chinese patients.

This study aimed to explore the relationships between EuroSCORE II, AVR status, and all-cause mortality in patients with moderate-to-severe AS, and to identify the optimal EuroSCORE II cut-off value for prognostic use in patients without AVR.

## 2. Methods

### 2.1 Study Design and Participants

This retrospective study included 1033 patients with moderate-to-severe AS from three heart valve centers (Second Affiliated Hospital of Shantou University Medical College, First Affiliated Hospital of Sun Yat-sen University, and Affiliated Hospital of Guangdong Medical University) between January 2014 and July 2023. The data were derived from the ARISTOTLE database. The study followed the Declaration of Helsinki and was approved by the Ethics Review Committee. Inclusion criteria were: (1) initial diagnosis of moderate-to-severe AS without severe dysfunction or malignancy of other organs; (2) no prior AVR history. Exclusion criterion was missing baseline data.

Patients were divided into high-risk and low-risk groups using a EuroSCORE II cut-off value of 4%, following the 2017 ESC/EACTS guidelines [15]. Four groups were created based on AVR and EuroSCORE II. The study was approved by the Ethics Review Committee of Shantou University Medical College, following the Declaration of Helsinki. Data were collected from 3 hospital records, and follow-up was via visits or calls. Verbal consent was obtained, approved by the Ethics Review Committee (ERB number: 2024-23).

### 2.2 Data Collection and Definitions

Patient characteristics (age, sex, height, body mass, smoking, and alcohol history) and preoperative comorbidities (hypertension, diabetes, extra-cardiac artery lesions, stroke, etc.) were validated by medical professionals using blood tests or imaging. AVR surgery details were obtained from electronic records or interviews. Laboratory tests within 24 hours of admission included (white blood cell (WBC), hemoglobin (Hb), creatinine, uric acid, cholesterol levels, etc.). Medication use (anti-platelet drugs, statins, β-blockers, diuretics, etc.) and echocardiographic indices (left ventricular ejection fraction (LVEF), atrioventricular maximum (AV-V max), aortic valve - mean gradient (AV-MG), aortic valve area (AVA), pulmonary arterial hypertension (PAH), etc.) were recorded. EuroSCORE II was calculated using a specific formula [11].

### 2.3 Definitions of Echocardiographic Indices

Transthoracic echocardiography followed American Society of Echocardiography guidelines [12]. AS severity was defined as [3, 4]: (1) moderate AS: 1 cm^2^ < AVA ≤ 1.5 cm^2^, 3 m/s < Vmax ≤ 3.9 m/s, or < 20 mmHg < MG ≤ 39 mmHg; and (2) severe AS: AVA ≤1 cm^2^, Vmax ≥4 m/s, or MG ≥40 mmHg. PAH was defined as a pulmonary arterial pressure ≥25 mmHg, according to the Guidelines for the Diagnosis and Treatment of Pulmonary Hypertension in China (2021 edition) [16].

### 2.4 Outcomes

The primary endpoint was all-cause mortality from the time of the diagnosis. The secondary endpoints were cardiovascular-related mortality and aortic stenosis-related mortality. The duration of follow-up was date of AS diagnosis to date of death or end of follow-up. And the trained medical staff collected the information through telephone contact with the participants or their families which was completed in July 2023.

### 2.5 Statistical Analysis

Continuous data were tested for normality using the Shapiro-Wilk method and presented as mean ± SD for normal distribution or median with inter-quartile range for skewed data. Categorical data were presented as counts and percentages. Homogeneous datasets were compared using ANOVA, while heterogeneous datasets were compared using the Kruskal-Wallis test. Differences between groups were evaluated using Student’s *t*-test or ANOVA for continuous data and Pearson’s chi-square test or Fisher’s exact test for categorical data, as appropriate. Kaplan-Meier analysis was used to calculate cumulative survival with the log-rank test. Univariate and multivariate Cox regression analyses were performed to generate hazard ratios (HR) and 95% confidence intervals (95% CI) for the relationship between confounders and all-cause mortality. Proportional hazards Cox regression models were adjusted for potential confounders in four models: Model 1 included smoking, alcohol, diabetes, atrial fibrillation (AF), hypertension, gout, systolic blood pressure, and body mass; Model 2 added WBC, Hb, platelet (PLT), low-density lipoprotein cholesterol (LDL-C), triglycerides (TG), total bilirubin (TBIL), N-terminal pro-B-type Natriuretic Peptide (NT-proBNP), troponin, and albumin; Model 3 added aortic valve deformity, rheumatic heart disease, AV-Vmax, AV-MG, AVA, mitral insufficiency, and mitral stenosis; Model 4 added medication use (anti-platelet agents, statins, angiotensin-converting enzyme inhibitors (ACEI), angiotensin receptor blockers (ARB), β-blockers, calcium channel blockers (CCB), diuretics, insulin, oral antidiabetic drugs, and oral anticoagulants). Considering the impact of different surgical methods on the all-cause mortality of people with aortic stenosis, we explored the effects of SAVR and TAVR, which are currently the mainstream surgical methods, on this population by Kaplan-Meier analysis and cox regression models. Subgroup analyses were conducted based on baseline sex, age (≤70 or >70 years), BMI (<24 or ≥24 kg/m^2^), diabetes, hypertension, coronary heart disease (CHD), and severity of AS (moderate or severe). The ROC curve was used to determine the optimal cut-off value of EuroSCORE II for predicting all-cause mortality. Multiple imputation via chained equations was used, and all analyses were performed using R software (version 4.4.1; The R Foundation for Statistical Computing, Vienna, Austria) and IBM SPSS Statistics (version 26; IBM Corporation, Armonk, NY, USA), with a two-sided *p* value ≤ 0.05 considered statistically significant.

## 3. Results

### 3.1 Baseline Characteristics 

**Fig. 1.**
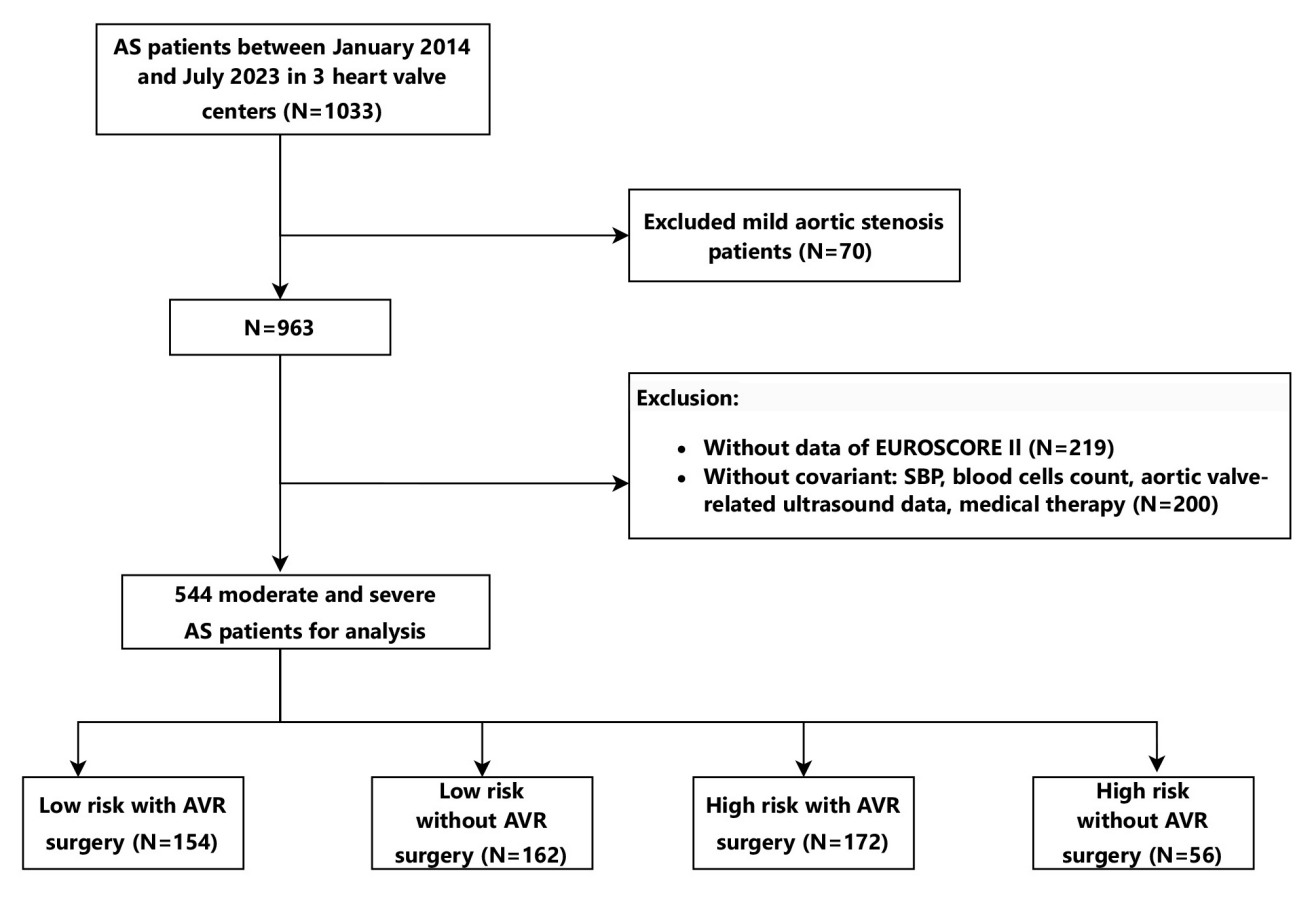
**Flow diagram describing the study sample**. The study was performed at three heart valve centers: the Second Affiliated Hospital of Shantou University Medical College, the First Affiliated Hospital of Sun Yat-sen University, and the Affiliated Hospital of Guangdong Medical University. Three heart valve centers are derived from a database called Aortic Valve Diseases RISk facTOr assessmenT and Prognosis modeL Construction (ARISTOTLE). 544 moderate-to-severe AS patients enrolled between January 2014 and July 2023 were allocated to four groups using the standard of cut-off value of 4% of EuroSCORE II dividing high-risk or low-risk and with/without AVR. AS, aortic valve stenosis; AVR, aortic valve replacement.

A total of 963 participants met the inclusion criteria. After excluding 419 patients without EuroSCORE II or baseline data, 544 participants (300 males, 244 females; mean age 66.00 years-old (interquartile range 57.00 to 74.00) were included in the study (Fig. 1, Table 1). Between the included (n = 544) and excluded (n = 489) cohorts, the difference was observed in the primary exposure, EuroSCORE II, potentially due to the extent of missing data. However, key covariates such as hypertension, diabetes, and chronic obstructive pulmonary disease (COPD) showed no significant differences (*p* > 0.05) (**Supplementary Table 1**). During a maximum 9.09-year follow-up (median follow-up 3.45 years), 117 participants (21.5%) reached the endpoint. Patients with a EuroSCORE II ≥4% who did not undergo AVR had significantly higher all-cause mortality rates compared to other groups (55.4% vs. 6.5%, 32.7%, and 13.4%; *p* < 0.001) (Table 1).

**Table 1.** **Clinical characteristics of the participants, stratified according to their EuroSCORE II and their AVR status**.

			Total	Low risk with AVR	Low risk without AVR	High risk with AVR	High risk without AVR	*p* value
			(N = 544)	(N = 154)	(N = 162)	(N = 172)	(N = 56)	
EuroSCORE II			3.35 (1.72–5.34)	2.38 (1.31–3.30)	1.72 (1.12–2.69)	5.95 (4.82–8.99)	5.34 (4.46–6.97)	<0.001
All-cause mortality (%)			117 (21.5)	10 (6.5)	53 (32.7)	23 (13.4)	31 (55.4)	<0.001
General characteristics								
	Hospital (%)							
		A	29 (5.33)	9 (5.84)	8 (4.94)	5 (2.91)	7 (12.50)	0.101
		B	454 (83.46)	124 (80.52)	138 (85.19)	146 (84.88)	46 (82.14)	
		C	61 (11.21)	21 (13.64)	16 (9.88)	21 (12.21)	3 (5.36)	
	Age (years)		66.00 (57.00–74.00)	61.50 (54.00–67.75)	69.00 (60.00–76.00)	64.00 (56.00–70.00)	78.00 (66.75–83.25)	<0.001
	Gender (%)							
		Male	300 (55.15)	105 (68.18)	92 (56.79)	79 (45.93)	24 (42.86)	<0.001
		Female	244 (44.85)	49 (31.82)	70 (43.21)	93 (54.07)	32 (57.14)	
	SBP (mmHg)		126.00 (111.00–140.00)	122.00 (112.00–135.00)	134.50 (119.00–149.00)	120.00 (106.75–135.00)	127.50 (112.00–148.75)	<0.001
	Weight (kg)		58.00 (50.00–65.00)	61.00 (52.00–67.00)	59.00 (53.00–65.00)	55.00 (48.22–62.78)	53.50 (47.75–62.25)	<0.001
State assessment								
	Status at the first clinic visit (%)							
		Elective	518 (95.22)	154 (100.00)	162 (100.00)	159 (92.44)	43 (76.79)	<0.001
		Urgent	26 (4.78)	0 (0.00)	0 (0.00)	13 (7.56)	13 (23.21)	
	The urgency of the surgery (%)							
		0	264 (48.53)	70 (45.45)	146 (90.12)	6 (3.49)	42 (75.00)	<0.001
		Non-CABG	276 (50.74)	84 (54.55)	16 (9.88)	164 (95.35)	12 (21.43)	
		2	3 (0.55)	0 (0.00)	0 (0.00)	2 (1.16)	1 (1.79)	
		3+	1 (0.18)	0 (0.00)	0 (0.00)	0 (0.00)	1 (1.79)	
	NYHA of first visit (%)							
		I	129 (23.71)	40 (25.97)	62 (38.27)	13 (7.56)	14 (25.00)	<0.001
		II	176 (32.35)	64 (41.56)	59 (36.42)	44 (25.58)	9 (16.07)	
		III	194 (35.66)	46 (29.87)	36 (22.22)	92 (53.49)	20 (35.71)	
		IV	45 (8.27)	4 (2.60)	5 (3.09)	23 (13.37)	13 (23.21)	
Comorbidities								
	Hypertension (%)		213 (39.15)	42 (27.27)	87 (53.70)	51 (29.65)	33 (58.93)	<0.001
	Rheumatic heart disease (%)		92 (16.91)	25 (16.23)	26 (16.05)	36 (20.93)	5 (8.93)	0.199
	Coronary disease (%)		113 (20.77)	31 (20.13)	43 (26.54)	29 (16.86)	10 (17.86)	0.157
	Previous major cardiac surgery (%)		210 (38.60)	60 (38.96)	0 (0.00)	142 (82.56)	8 (14.29)	<0.001
	Atrial fibrillation (%)		88 (16.18)	17 (11.04)	27 (16.67)	28 (16.28)	16 (28.57)	0.025
	Stroke (%)		36 (6.62)	6 (3.90)	18 (11.11)	6 (3.49)	6 (10.71)	0.010
	CKD (%)		33 (6.07)	4 (2.60)	11 (6.79)	10 (5.81)	8 (14.29)	0.018
	Extra-cardiac artery lesions (%)		33 (6.07)	1 (0.65)	12 (7.41)	9 (5.23)	11 (19.64)	<0.001
	COPD (%)		22 (4.04)	4 (2.60)	8 (4.94)	3 (1.74)	7 (12.50)	0.003
	Diabetes mellitus (%)		82 (15.07)	18 (11.69)	33 (20.37)	17 (9.88)	14 (25.00)	0.005
	History of gout (%)		20 (3.68)	1 (0.65)	10 (6.17)	3 (1.74)	6 (10.71)	0.001
	History of smoking (%)		149 (27.39)	50 (32.47)	43 (26.54)	42 (24.42)	14 (25.00)	0.395
	History of drinking (%)		84 (15.44)	26 (16.88)	25 (15.43)	27 (15.70)	6 (10.71)	0.750
Laboratory parameters								
	Hb (g/L)		126.00 (113.00–139.00)	132.50 (119.00–143.75)	126.00 (114.00–138.75)	126.00 (109.00–138.00)	116.00 (104.00–125.00)	<0.001
	WBC (10^9^/L)		6.96 (5.58–8.49)	7.03 (5.76–8.27)	6.86 (5.54–8.53)	6.93 (5.56–8.67)	6.86 (5.59–8.63)	0.958
	TC (mmol/L)		4.53 (3.80–5.10)	4.53 (4.00–5.30)	4.53 (3.82–5.40)	4.53 (3.70–4.70)	4.14 (3.42–4.62)	0.010
	HDL-C (mmol/L)		1.14 (0.95–1.28)	1.14 (0.99–1.27)	1.14 (0.94–1.36)	1.14 (0.95–1.19)	1.13 (0.93–1.32)	0.767
	LDL-C (mmol/L)		2.87 (2.34–3.26)	2.87 (2.45–3.45)	2.87 (2.38–3.44)	2.87 (2.33–2.89)	2.57 (2.01–3.02)	0.013
	TG (mmol/L)		1.23 (0.86–1.40)	1.27 (0.91–1.43)	1.21 (0.84–1.53)	1.27 (0.84–1.27)	1.06 (0.77–1.27)	0.024
	Cr (µmol/L)		82.00 (69.00–100.00)	78.90 (68.00–91.97)	81.00 (69.00–98.50)	83.50 (69.00–104.25)	98.55 (81.25–124.25)	<0.001
	eGFR (mL/min/1.73 m^2^)		59.30 (44.53–77.61)	71.89 (57.67–90.22)	58.87 (46.81–74.62)	56.86 (42.85–74.01)	39.78 (30.03–47.90)	<0.001
	UREA (mmol/L)		6.30 (4.90–8.00)	6.20 (4.82–7.15)	6.15 (4.80–7.68)	6.45 (4.90–8.33)	7.66 (5.95–10.57)	0.001
	TBIL (µmol/L)		12.80 (9.70–18.20)	12.50 (9.72–16.30)	11.50 (8.83–15.35)	13.80 (10.50–19.73)	13.55 (10.45–23.08)	0.004
	Blood glucose (mmol/L)		5.00 (4.40–5.80)	4.90 (4.30–5.44)	5.00 (4.30–5.84)	4.90 (4.50–5.93)	5.25 (4.70–6.16)	0.037
	Uric acid (µmol/L)		411.00 (325.00–494.15)	404.35 (328.50–468.65)	402.90 (303.50–486.75)	416.36 (321.75–533.72)	447.50 (359.00–511.25)	0.104
	Elevated NT-proBNP (%)		275 (50.55)	64 (41.56)	91 (56.17)	80 (46.51)	40 (71.43)	<0.001
	Troponin40 (%)		15 (2.76)	6 (3.90)	2 (1.23)	4 (2.33)	3 (5.36)	0.299

Continuous datasets were tested for normality using the Shapiro–Wilk method, and the data are presented as mean ± SD for normal distribution, or median and interquartile range for skewed data. Categorical data are presented as counts and percentages (%). Homogeneous datasets compared using ANOVA, heterogeneous using Kruskal–Wallis test. Student’s *t*-test or ANOVA was used to evaluate differences between groups with respect to continuous data, and Pearson’s chi-square test or Fisher’s exact test was used to compare categorical datasets, as appropriate. Group 1 (low risk (EuroSCORE II index <4%) with AVR); Group 2 (low risk (EuroSCORE II index <4%) without AVR); Group 3 (high risk (EuroSCORE II index ≥4%) with AVR); Group 4 (high risk (EuroSCORE II index ≥4%) without AVR). *p* ≤ 0.05 was considered statistically significant. *p* > 0.05, no significant. SBP, systolic blood pressure; CABG, coronary artery bypass grafting; COPD, chronic obstructive pulmonary disease; Hb, haemoglobin; eGFR, estimated glomerular filtration rate; UREA, urea nitrogen; NT-proBNP, N-Terminal pro-Brain Natriuretic Peptide; WBC, white blood cell; TC, total cholesterol; HDL-C, high-density lipoprotein-cholesterol; LDL-C, lowdensity lipoprotein-cholesterol; TG, triglyceride; Cr, creatinine; TBIL, total bilirubin; CKD, chronic kidney disease.

Participants were divided into two groups based on EuroSCORE II: low-risk (<4%) and high-risk (≥4%). High-risk patients were more likely to be male require urgent surgery, have severe cardiac dysfunction, and other mitral or aortic valve diseases (**Supplementary Table2**).

When divided into four groups (Group 1: low risk with AVR; Group 2: low risk without AVR; Group 3: high risk with AVR; Group 4: high risk without AVR), Group 4 patients were older, had lower weight and higher NYHA scores, more comorbidities (hypertension, AF, stroke, chronic kidney disease (CKD), extra-cardiac artery lesions, COPD, diabetes), higher levels of creatinine (Cr), total bilirubin (TBIL), and blood glucose, and lower levels of LDL-C and TG (all *p* ≤ 0.05) (Table 1), which was closely with higher EuroSCORE II values and were independently associated with increased all-cause mortality (Table 2). They also had higher incidence of other valvular diseases, severe PAH, and lower LVEF (**Supplementary Table 3**), and higher use of statins, insulin, and oral anticoagulants (*p* > 0.05).

**Table 2.** **Relationships of EuroSCORE II and AVR status with the all-cause mortality of the participants**.

Variables	Low risk with surgery	Low risk without surgery	High risk with surgery	High risk without surgery	*p* value
	(N = 154)	(N = 162)	(N = 172)	(N = 56)	
Model 1	Reference	5.541 (2.737–11.216)	1.899 (0.896–4.025)	10.058 (4.632–21.840)	<0.001
Model 2	Reference	4.211 (2.053–8.637)	1.450 (0.673–3.125)	8.168 (3.704–18.015)	<0.001
Model 3	Reference	4.064 (1.958–8.433)	1.321 (0.605–2.887)	7.342 (3.281–16.427)	<0.001
Model 4	Reference	3.492 (1.583–7.703)	1.333 (0.605–2.936)	6.605 (2.817–15.486)	<0.001

Further adjustment for potential confounders in Model 1–4 (Table 2). The fully adjusted HR and 95% CI in Model 4 for groups 2–4 vs. group 1 were 3.492 (1.583–7.703), 1.333 (0.605–2.936), and 6.605 (2.817–15.486), respectively (*p* < 0.05). *p* ≤ 0.05 was considered statistically significant. *p* > 0.05, no significant. Model 1 was adjusted for smoking status, alcohol consumption status, diabetes, atrial fibrillation, hypertension, gout, systolic blood pressure, and body mass; Model 2 was adjusted for the parameters in model 1, with the addition of WBC, RBC, PLT, LDLC, TG, TBIL, NTproBNP, troponin, and albumin; Model 3 was adjusted for the parameters in model 2, plus aortic valve deformity, rheumatic heart disease, AV-Vmax, AV-MG, the degree of AVA mitral insufficiency, and mitral stenosis; Model 4 was adjusted for the parameters in Model 3, with the addition of anti-platelet agent, statin, ACEI, ARB, β-blocker, CCB, diuretic, insulin, oral antidiabetic drug, and oral anticoagulant use. AV-Vmax, maximum flow velocity through the aortic valve; AV-MG, mean gradient across the aortic valve; AVA, aortic valve area; ACEI, angiotensin converting enzyme inhibitor; ARB, angiotensin receptor blocker; CCB, calcium channel blocker; RBC, red blood cell; PLT, platelet.

### 3.2 Relationships of All-Cause Mortality With EuroSCORE II and AVR Status

**Fig. 2.**
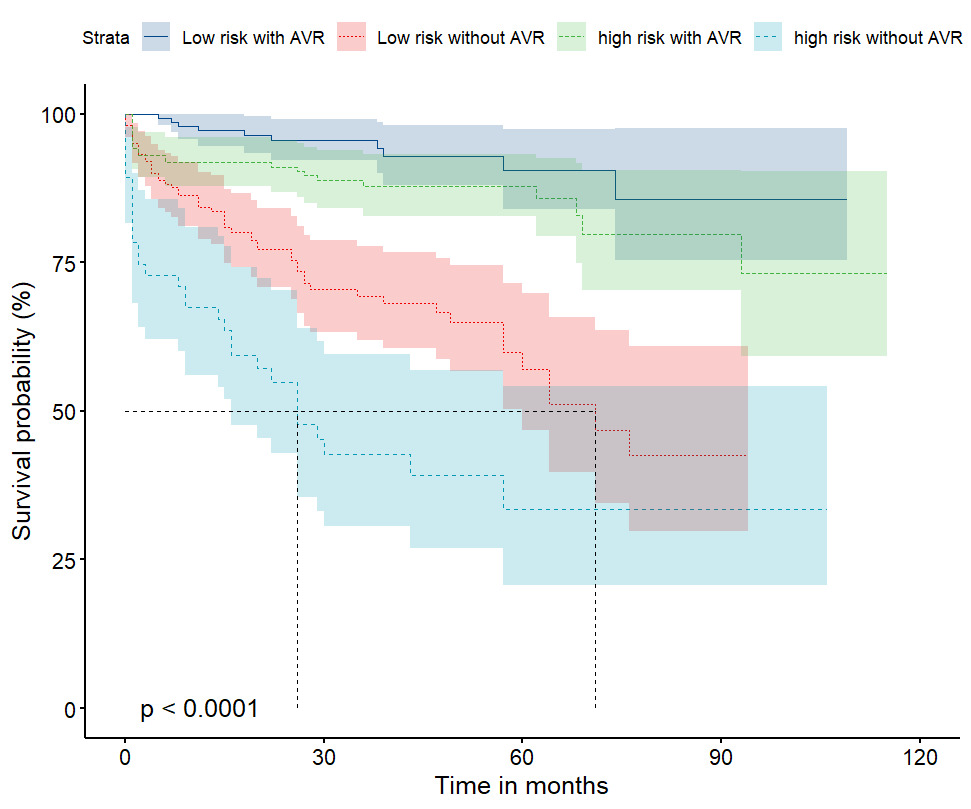
**Kaplan–Meier survival curves for the participants, categorized according to their EuroSCORE II and their AVR status (total 544 participants)**. The analysis demonstrated that the participants in the group with high EuroSCORE II and who had not undergone AVR were at the highest risk of all-cause mortality of the four groups (log-rank *p* < 0.001).

When divided by EuroSCORE II in patients without AVR, the high-risk group had higher all-cause mortality than the low-risk group (*p* < 0.001) (**Supplementary Fig. 1**), while no difference was found in participants with AVR (*p* = 0.067) (**Supplementary Fig. 2**). When stratified by both EuroSCORE II and AVR status, Group 4 (high risk without AVR) had the highest all-cause mortality (*p* < 0.001) (Fig. 2). In the low-risk group, those without AVR had poorer cumulative survival (*p* < 0.001) (**Supplementary Fig. 3**), and the same was observed in the high-risk group (*p* < 0.001) (**Supplementary Fig. 4**). No difference was found between those who underwent AVR (Whether it is SAVR or TAVR) (*p* = 0.69) (**Supplementary Fig. 5**).

### 3.3 Predictors of All-Cause Mortality

Univariate regression analysis (**Supplementary Table 4**) identified age, coronary artery disease (CAD), CKD, COPD, diabetes, AF, hypertension, albumin, critical status, urgency of surgery, LVEF, pulmonary hypertension, statin use, ACEI/ARB use, and insulin use as predictors of all-cause mortality, with Group 2 and Group 4 exhibiting a 5.97-fold (HR 5.966, 95% CI 3.033–11.737, *p* < 0.001) and 12.17-fold (HR 12.168, 95% CI 5.958–24.848, *p* < 0.001) higher risk of mortality compared to Group 1, respectively. However, no significant difference was observed between Group 3 and Group 1 (HR 1.972, 95% CI 0.938–4.144, *p* = 0.073). Multivariate Cox regression analyses yielded similar results, with Group 2 and Group 4 demonstrating a 3.71-fold (HR 3.705, 95% CI 1.738–7.896, *p* = 0.001) and 6.89-fold (HR 6.891, 95% CI 3.083–15.401, *p* < 0.001) higher risk of mortality compared to Group 1, respectively. For the Cox regression models (Table 2), Model 4 revealed that, with Group 1 as the reference, the risk of mortality in Group 2, Group 3, and Group 4 increased significantly (HR 3.492, 95% CI 1.583–7.703; HR 1.333, 95% CI 0.605–2.936; HR 6.605, 95% CI 2.817–15.486, respectively). The further regression model also revealed that there was no significant difference between SAVR and TAVR on all-cause mortality of the population of moderate-to-severe aortic stenosis (*p* = 0.848) (**Supplementary Table 5**). Additionally, low-risk participants with AVR were associated with a 0.23-fold risk (HR 0.230, 95% CI 0.118–0.857, *p* < 0.01) compared to those without AVR (**Supplementary Table 6**).

### 3.4 Results of the Subgroup Analyses

**Fig. 3.**
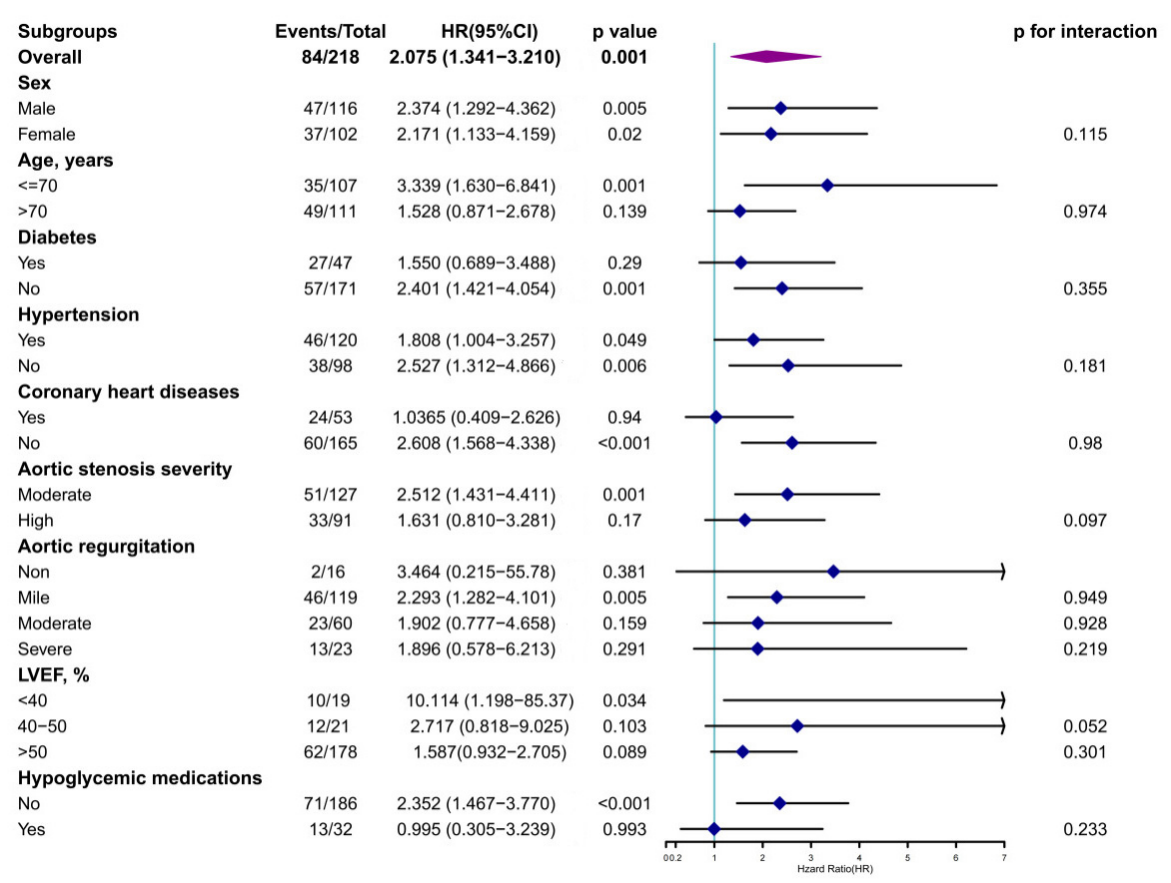
**Results of the subgroup analysis of the association between EuroSCORE II and the lack of AVR**. Further subgroup analyses were performed after the stratification of the participants according to baseline sex, age (≤70 or >70 years), BMI (<24 or ≥24 kg/m^2^), diabetes, hypertension, CHD, and the severity of AS (moderate or severe), to assess the consistency of the prognostic use of EuroSCORE II for all-cause mortality. HR, hazard ratio; CI, confidence interval.

Fig. 3 shows a subgroup analysis of all-cause mortality. In patients without AVR, EuroSCORE II was correlated with mortality in younger (≤70 years), male, lacking diabetes, hypertension, or CHD, not taking hypoglycemic medications, with mild aortic regurgitation, LVEF <40%, and moderate AS (*p* < 0.05). This association remained significant after adjustment, with no significant interactions (*p* > 0.05). For patients with AVR, EuroSCORE II was similarly associated with mortality in those without diabetes or CHD and not taking hypoglycemic medications, with no significant interactions (*p* > 0.05) (**Supplementary Fig. 6**).

### 3.5 Optimal Cut-Off Value of EuroSCORE II for the Prediction of Outcomes in the Participants Who had not Performed AVR

**Fig. 4.**
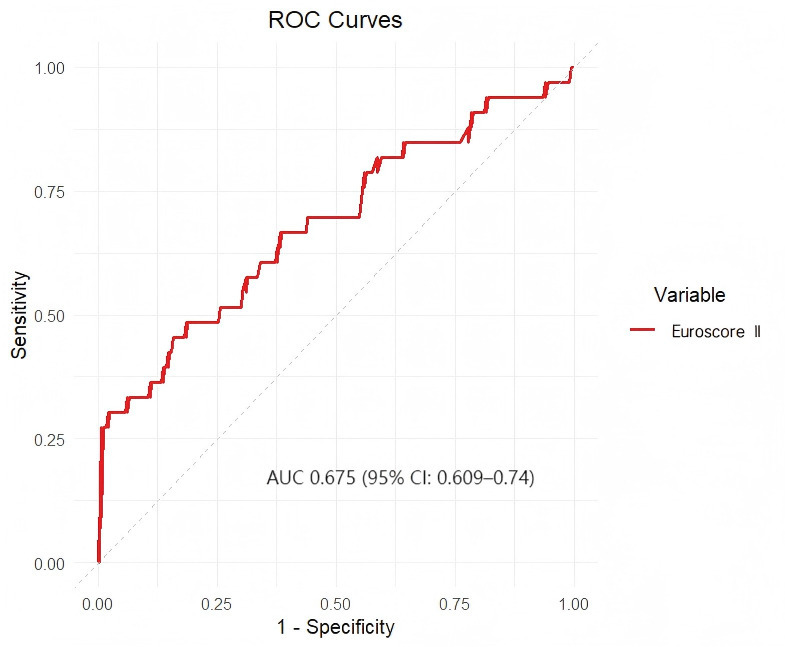
**ROC curve for the use of EuroSCORE II to evaluate the prognosis of the participants who had not undergone AVR**. The optimal cut-off value of EuroSCORE II identified for the prediction of the primary outcome in the participants who had not undergone AVR was 2.23%, which was associated with an AUC of 0.675 (95% CI: 0.609–0.74).

For the participants who had not performed AVR, the optimal cut-off value of EuroSCORE II identified for predicting the outcome was 2.23% (AUC 0.675, 95% CI 0.609–0.74, *p* < 0.001) (Fig. 4). The participants whose EuroSCORE II index ≥2.23% had a 2.111 (HR 2.111, 95% CI 1.069–4.166, *p* = 0.031) -fold higher risk of all-cause mortality than those <2.23% (**Supplementary Fig. 7**, **Supplementary Table 7**).

## 4. Discussion

A growing body of evidence demonstrates that patients with symptomatic severe AS have a poor prognosis if they do not undergo surgery, with a mean survival time of <4 years [8]. Therefore, AVR is widely recommended for the treatment of such patients [17]. EuroSCORE II, originally developed for perioperative risk stratification in cardiac surgery, incorporates variables such as age, renal function, and extracardiac arteriopathy, which are also implicated in the progression of calcific aortic valve disease [6].

The study found that high-risk group (EuroSCORE II ≥4%) was associated with higher risk of all-cause mortality. Our baseline results revealed that a high EuroSCORE II reflects not only heart disease severity but also broader end-organ damage, including key factors like kidney dysfunction, anemia, liver congestion from heart failure, and malnutrition.

It is this comprehensive physiological decline that renders patients too vulnerable to withstand the persistent hemodynamic stress of untreated severe aortic stenosis, ultimately leading to the observed extremely high mortality [18]. The pathophysiological overlap between cardiovascular risk factors and AS progression—particularly the roles of chronic inflammation, endothelial dysfunction, and calcification [19]—suggests that EuroSCORE II may be used for not only surgical risk but also underlying disease severity and systemic vascular health.

While the STS score is another widely used risk model, a meta-analysis of 22 studies involving 145,592 cardiac surgery procedures showed that the overall discriminatory ability of the EuroSCORE II is good and similar to that of the STS risk score. However, the STS risk score has shown a suboptimal calibration in some validation studies, particularly in European cardiac surgical populations [20]. Future studies directly comparing these models in Chinese patients with AS would be valuable.

In this context, our study extends the utility of EuroSCORE II beyond the perioperative setting, demonstrating its prognostic value for long-term mortality within the past 10 years in Chinese patients with moderate-to-severe AS, regardless of AVR status. We found that patients in the high-risk group (EuroSCORE II ≥4%), irrespective of whether they underwent AVR, exhibited a significantly higher risk of all-cause mortality compared to those in the low-risk group, which was align with previous studies linking EuroSCORE II to outcomes in TAVI patients [16, 21, 22]. A retrospective study of 350 patients with moderate-to-severe AS undergoing TAVI found that 30-day mortality was associated with higher EuroSCORE II (12.6 ± 1.8% vs. 7.5 ± 0.3%, *p* < 0.001) and an AUC of 0.70, indicating its predictive value for short-term mortality [16]. Another study of 59 patients with severe symptomatic AS who underwent TAVI between 2010 and 2014 also showed that EuroSCORE II predicts in-hospital and 30-day mortality [23]. However, the short duration of follow-up in these studies limits their relevance to clinical practice. In contrast, a long-term retrospective study by Fan *et al*. [24] categorized 332 patients with low-gradient severe AS and preserved LVEF into high-risk (EuroSCORE II ≥4%, N = 115) and low-risk (EuroSCORE II <4%, N = 208) groups and followed them for 2 years [25]. Unlike these reports that were limited to follow-up time, our long-term follow-up offers similar insights that the high-risk group had significantly poorer cumulative survival compared to the low-risk group when using 4% as a cut-off value.

Moreover, moderate-to-severe aortic stenosis patients with a low-risk EuroSCORE II (<4%) were also recommended to undergo AVR, as it significantly reduced the risk by 77% of all-cause mortality, which was similar to some major trials in low-risk patients.

Notably, the PARTNER2 study [26] and the SURTAVI study [27] using TAVR in low-risk patients performed by the American College of Cardiology (ACC), including the PARTNER 3 study (using the SAPIEN-3 ultra transcatheter heart valve) and the Evolut Low Risk Trial study (using the self-expanding Evolute R valve) [28] showed TAVR’s non-inferiority or superiority to SAVR in short to medium-term outcomes. After 5 years of follow-up, the PARTNER 3 study revealed similar annual rates of cardiovascular mortality, stroke, and re-hospitalization for both TAVR and SAVR (approximately 1%, 1%, and 3%, respectively). These findings support recommending either procedure for low EuroSCORE II patients, offering benefits such as shorter hospital stays and lower mortality risks [29]. Therefore, surgical or transcatheter AVR (SAVR/TAVI) is recommended for low-risk patients to achieve a clear survival benefit, aligning with international guidelines and providing real-world validation in a Chinese population. Our study confirms that SAVR and TAVI have similar outcomes for moderate-to-severe AS. The prospective NOTION trial (N = 280) showed no significant differences in 2-year all-cause (8.0% vs. 9.8%; *p* = 0.54) or cardiovascular mortality (6.5% vs. 9.1%; *p* = 0.40) between TAVR and SAVR [30]. Moreover, the NOTION trial’s extended data revealed no significant all-cause mortality difference over 8 years [15]. The 2021 ESC/EACTS guidelines recommend SAVR for patients under 75, and TAVR for those over 75, high-risk (STS-PROM or EuroSCORE II >8%), or ineligible for surgery (class I) [9]. Despite guidelines, numerous randomized clinical trials show TAVR is at least as effective as SAVR for short- to medium-term results [31, 32].

A key validation study demonstrated EuroSCORE II’s inaccuracies in predicting mortality for cardiac surgery patients, either underestimating for high-risk or overestimating for low-risk groups [32]. Our ROC analysis identified a EuroSCORE II cut-off of 2.23% (AUC, 0.675) for moderate-to-severe AS patients without AVR, validated by Kaplan-Meier (log-rank *p* < 0.001) and Cox analyses (HR 2.111, 95% CI 1.069–4.166, *p* = 0.031). This threshold, more accurate than the traditional 4%, may enhance clinical detection, treatment response, and prognosis for these patients. Notably, the original 4% value applied broadly to AS patients, regardless of surgery, whereas our 2.23% value is specific to those with moderate-to-severe AS who have not undergone AVR.

The study’s strengths include its multi-centre observational design with verifiable external validity, a 9-year follow-up period, and robust adjustment for confounders, confirming the significant association between EuroSCORE II and all-cause mortality in moderate-to-severe AS patients. These findings enrich the clinical utility of EuroSCORE II in predicting AS outcomes, potentially improving patient outcomes and reducing disease burden.

Our study has the following strengths: firstly, our research uniquely examines the predictive value of EUROSCORE II for both moderate and severe aortic stenosis (AS) patients, expanding the scope beyond the commonly studied severe AS population and providing insights into a broader spectrum of disease severity. Secondly, our study is based on multi-center data sourced from three centers within a database jointly collected by multiple Chinese valve centers, ensuring the diversity and representativeness of the sample. Additionally, our study benefits from a long follow-up period of up to 9.09 years, with a wide-ranging span that enhances the stability and reliability of our conclusions. Importantly, we innovatively propose modifying the EUROSCORE II cut-off value to 2.23% for moderate-to-severe AS patients without AVR surgery, addressing a gap in the literature and offering a novel perspective on risk stratification.

However, limitations exist. This study is a Chinese-based retrospective cohort which has its inherent selection bias and our sample size was relatively limited, thus lacking generalization and necessitating external validation. Additionally, the applicability of EuroSCORE II is limited by etiological differentiation (e.g., degenerative calcific vs. rheumatic) for aortic stenosis due to lacking historical records data. Moreover, echocardiographic and laboratory parameters were static, risking bias from measurement errors or inter-center criteria differences.

Finally, though EuroSCORE II was associated with mortality without AVR, its modest accuracy demands supplementation with clinical and imaging data for reliable risk stratification.

## 5. Conclusions

In conclusion, both the EuroSCORE II (cut-off value 4%) and AVR status were independently associated with the long-term prognosis of patients with moderate-to-severe AS. Patients with a low-risk EuroSCORE II (<4%) should be recommended to undergo AVR (either SAVR or TAVI). Furthermore, our analysis identified an exploratory, lower EuroSCORE II cutoff value of 2.23% for risk stratification in patients who did not undergo AVR. However, this novel threshold is derived from retrospective data and requires prospective validation before any clinical application can be considered.

## Data Availability

Our heart valvular disease intervention center construction unit from The Second Affiliated Hospital of Shantou University Medical College is part of the National Clinical Medical Research Center for Radiology and Therapy, Interventional Center for Valvular Disease, Dongfang Huaxia Cardiovascular Health Institute, Suzhou Industrial Park. Our data were from Three heart valve centers, which are derived from a database called Aortic Valve Diseases RISk facTOr assessmenT and Prognosis modeL Construction (ARISTOTLE). The datasets used and analyzed during the current study are available from the corresponding authors upon reasonable request.

## Abbreviations

EuroSCORE II, European System for Cardiac Operative Risk Evaluation; SBP, systolic blood pressure; CKD, chronic kidney disease; COPD, chronic obstructive pulmonary disease; HB, haemoglobin; TC, total cholesterol; HDL-C, high-density lipoprotein-cholesterol; LDL-C, low-density lipoprotein-cholesterol; TG, triglyceride; Cr, creatinine; CC, creatinine clearance; TBIL, total bilirubin; LVEF, left ventricular ejection fraction; AV-Vmax, maximum velocity of blood flow through the aortic valve; AV-MG, mean gradient across the aortic valve; AVA, aortic valve area; PAH, pulmonary arterial hypertension; ACEI, angiotensin converting enzyme inhibitor; ARB, angiotensin receptor blocker; CCB, calcium channel blocker.

## Supplementary Material

Supplementary material associated with this article can be found, in the online version, at https://doi.org/10.31083/RCM42757.
