# Supplementary material for: Evaluation of EuroSCORE II to Determine the Prognosis of Patients With Moderate-To-Severe Aortic Stenosis: A Long-Term Retrospective Study
Source: Rev Cardiovasc Med. 2026 Feb 4;27(2):42757. doi: 10.31083/RCM42757 (PMC12960013; doi:10.31083/RCM42757)
Supplement: Supplementary file 1 [file 2153-8174-27-2-42757-s1.zip › Supplementary Material.docx]

**Supplementary Table 1.** comparison of baseline characteristics between the included cohort (n=544) and excluded patients due to missing data (n=489).

|  | Total(N=1033) | Excluded  patients  (n=489) | Included cohort (n=544) | P value |  |
| --- | --- | --- | --- | --- | --- |
| EUROSCORE II | 3.10 (1.63-5.05) | 2.53 (1.58-4.15) | 3.35 (1.72-5.34) | 0.002 |  |
| **General characteristics** | | | | |  |
| Hospital (%) |  |  |  |  |  |
| A | 91 (8.81) | 62 (12.68) | 29 (5.33) | <0.001 |  |
| B | 640 (61.96) | 186 (38.04) | 454 (83.46) | |  |
| C | 302 (29.24) | 241 (49.28) | 61 (11.21) | |  |
| Age (years-old) | 67.50 (59.00-77.00) | 70.50 (62.00-79.00) | 66.00 (57.00-74.00) | 0.067 |  |
| Gender (%) |  |  |  |  |  |
| Male | 537 (52.29) | 237 (49.07) | 300 (55.15) | 0.052 |  |
| Female | 490 (47.71) | 246 (50.93) | 244 (44.85) | |  |
| Missed value | 6 (0.58) | 6 (1.23) | 0 (0.00) | |  |
| SBP (mmHg) | 127.00 (112.00-143.00) | 130.00 (112.00-146.50) | 126.00 (111.00-140.00) | 0.039 |  |
| Weight (kg) | 57.95 (50.00-65.00) | 57.85 (50.00-65.00) | 58.00 (50.00-65.00) | 0.658 |  |
| **State assessment** |  |  |  |  |  |
| Status at the first clinic visit (%) | | | | |  |
| elective | 985 (95.35) | 467 (95.50) | 518 (95.22) | 0.831 |  |
| urgent | 48 (4.65) | 22 (4.50) | 26 (4.78) | |  |
| The urgency of the surgery (%) | | | | |  |
| 0 | 435 (42.11) | 171 (34.97) | 264 (48.53) | <0.001 |  |
| non-CABG | 580 (56.15) | 304 (62.17) | 276 (50.74) | |  |
| 2 | 13 (1.26) | 10 (2.04) | 3 (0.55) |  |  |
| 3+ | 5 (0.48) | 4 (0.82) | 1 (0.18) |  |  |
| NYHA of first visit (%) | | | | |  |
| I | 247 (23.91) | 118 (24.13) | 129 (23.71) | 0.034 |  |
| II | 314 (30.40) | 138 (28.22) | 176 (32.35) | |  |
| III | 360 (34.85) | 166 (33.95) | 194 (35.66) | |  |
| IV | 112 (10.84) | 67 (13.70) | 45 (8.27) | |  |
| **Comorbidities** |  |  |  |  |  |
| Hypertension (%) | 412 (39.88) | 199 (40.70) | 213 (39.15) | 0.614 |  |
| Active endocarditis (%) | 20 (1.94) | 8 (1.64) | 12 (2.21) | 0.507 |  |
| Previous major cardiac surgery (%) | 268 (25.94) | 58 (11.86) | 210 (38.60) | <0.001 |  |
| Atrial fibrillation (%) | 146 (14.13) | 58 (11.86) | 88 (16.18) | 0.047 |  |
| Stroke (%) | 82 (7.94) | 46 (9.41) | 36 (6.62) | 0.098 |  |
| CKD (%) | 65 (6.35) | 33 (6.75) | 33 (6.07) | 0.654 |  |
| Extra-cardiac artery lesions (%) | 58 (5.61) | 25 (5.11) | 33 (6.07) | 0.506 |  |
| COPD (%) | 41 (3.97) | 19 (3.89) | 22 (4.04) | 0.896 |  |
| Diabetes mellitus (%) | 152 (14.71) | 70 (14.31) | 82 (15.07) | 0.731 |  |
| History of gout (%) | 52 (5.03) | 32 (6.54) | 20 (3.68) | 0.035 |  |
| History of smoking (%) | 242 (23.43) | 93 (19.02) | 149 (27.39) | 0.002 |  |
| History of drinking (%) | 124 (12.00) | 40 (8.18) | 84 (15.44) | <0.001 |  |
| Laboratory parameters |  |  |  |  |  |
| HB (g/L) | 125.00 (110.00-138.00) | 122.50 (108.00-135.68) | 126.00 (113.00-139.00) | 0.010 |  |
| WBC (10^9^/L) | 7.12 (5.71-8.84) | 7.40 (5.90-9.20) | 6.96 (5.58-8.49) | 0.005 |  |
| PLT (10^9^/L) | 197.00 (157.00-238.00) | 196.50 (155.00-234.00) | 199.50 (160.00-243.00) | 0.104 |  |
| TC (mmol/L) | 4.30 (3.59-5.20) | 4.21 (3.50-5.12) | 4.40 (3.66-5.30) | 0.067 |  |
| HDL-C (mmol/L) | 1.10 (0.92-1.34) | 1.09 (0.92-1.31) | 1.11 (0.92-1.35) | 0.548 |  |
| LDL-C (mmol/L) | 2.66 (2.10-3.37) | 2.58 (2.02-3.31) | 2.74 (2.20-3.42) | 0.013 |  |
| TG (mmol/L) | 1.04 (0.79-1.43) | 1.00 (0.80-1.33) | 1.07 (0.79-1.50) | 0.034 |  |
| eGFR (mL/min/1.73m^2^) | 57.66 (43.23-74.57) | 54.42 (40.32-69.23) | 59.30 (44.53-77.60) | 0.002 |  |
| UREA (mmol/L) | 6.30 (5.00-8.30) | 6.30 (5.10-8.80) | 6.30 (4.90-8.00) | 0.141 |  |
| TBIL (mmol/L) | 12.90 (9.30-18.10) | 13.00 (8.90-18.00) | 12.80 (9.70-18.20) | 0.502 |  |
| Blood glucose (mmol/L) | 5.00 (4.40-5.90) | 5.00 (4.46-6.00) | 5.00 (4.40-5.80) | 0.205 |  |
| Uric acid (mmol/L) | 400.00 (312.92-489.65) | 396.50 (305.03-481.22) | 406.00 (322.00-499.00) | 0.095 |  |
| Elevated NT-proBNP (%) | 597 (57.79) | 322 (65.85) | 275(50.55) | <0.001 |  |
| Troponin＞40 (%) | 38 (3.68) | 23 (4.70) | 15 (2.76) | 0.135 |  |
| Echocardiogram |  |  |  |  |  |
| LVEF (%) | 61.00 (52.00-69.00) | 58.00 (49.00-65.00) | 65.00 (56.00-72.00) | <0.001 |  |
| Aortic valve bilobate malformation (%) | 118 (11.42) | 35 (7.16) | 83 (15.26) | <0.001 |  |
| AV-Vmax (m/s) | 4.16 (3.50-4.78) | 4.00 (3.30-4.70) | 4.20 (3.72-4.79) | <0.001 |  |
| AV-MG (mmHg) | 41.00 (29.00-54.00) | 36.00 (24.00-51.00) | 42.50 (33.00-55.25) | <0.001 |  |
| AVA (cm^2^) | 0.87 (0.70-1.10) | 0.90 (0.66-1.20) | 0.84 (0.70-1.00) | 0.004 |  |
| PAH (%) |  |  |  |  |  |
| 0-30mmHg | 512 (49.56) | 289 (59.10) | 223 (40.99) | <0.001 |  |
| 31-55mmHg | 409 (39.59) | 151 (30.88) | 258 (47.43) | |  |
| >55mmHg | 112 (10.84) | 49 (10.02) | 63 (11.58) | |  |
| Aortic regurgitation (%) |  |  |  |  |  |
| Non | 72 (6.97) | 35 (7.16) | 37(6.80) | 0.600 |  |
| Mild | 505 (48.89) | 249 (50.92) | 256 (47.06) | |  |
| Moderate | 311 (30.11) | 140 (28.63) | 171 (31.43) | |  |
| Severe | 145 (14.04) | 65 (13.29) | 80 (14.71) | |  |
| Aortic stenosis (%) |  |  |  |  |  |
| Non | 11 (1.06) | 11 (2.25) | 0 (0.00) | <0.001 |  |
| Mild | 83 (8.03) | 83 (16.97) | 0 (0.00) |  |  |
| Moderate | 387 (37.46) | 157 (32.11) | 230 (42.28) |  |  |
| Severe | 552 (53.44) | 238 (48.67) | 314 (57.72) | |  |
| Mitral regurgitation (%) |  |  |  |  |  |
| Non | 213(20.62) | 93 (19.02) | 120 (22.06) | 0.351 |  |
| Mild | 523 (50.63) | 244 (49.90) | 279 (51.29) | |  |
| Moderate | 213 (20.62) | 107 (21.88) | 106 (19.49) | |  |
| Severe | 84 (8.13) | 45 (9.20) | 39 (7.17) |  |  |
| Mitral stenosis (%) |  |  |  |  |  |
| Non | 785 (76.19) | 383 (72.61) | 402 (73.90) | 0.314 |  |
| Mild | 85 (8.23) | 34 (8.79) | 51 ((9.38) |  |  |
| Moderate | 86 (8.33) | 40 (10.34) | 46 (8.46) | |  |
| Severe | 77 (7.45) | 32 (8.27) | 45 (8.27) | |  |
| **Cardiovascular medical therapy** | | | | |  |
| Anti-plate medicine (%) | 414 (40.08) | 208 (42.54) | 206 (37.87) | 0.126 |  |
| Stains (%) | 435 (42.11) | 235 (48.06) | 200 (36.76) | <0.001 |  |
| ACEI/ARB (%) | 239 (23.14) | 97 (19.84) | 142 (26.10) | 0.017 |  |
| β-receptor blockers (%) | 493 (47.73) | 207 (42.33) | 286 (52.57) | 0.001 |  |
| CCB (%) | 168 (16.26) | 87 (17.79) | 81 (14.89) | 0.207 |  |
| Diuretic drug (%) | 709 (68.64) | 325 (66.46) | 384 (70.59) | 0.154 |  |
| Insulin (%) | 73 (7.07) | 26 (5.32) | 47 (8.64) | 0.037 |  |
| Oral antidiabetic drug (%) | 149 (14.42) | 76 (15.54) | 73 (13.42) | 0.332 |  |
| Oral anticoagulants (%) | 393 (38.04) | 136 (27.81) | 257 (47.24) | <0.001 |  |

Continuous variables: median (IQR) or Mean ± Standard Deviation; categorical variables: N (%). P-values for categorical variables were derived from Pearson chi-square tests. *P* ≤0.05 was considered statistically significant. *P* >0.05, no significant.

A, Second Affiliated Hospital of Shantou University Medical College, B, the First Affiliated Hospital of Sun Yat-sen University, C, the Affiliated Hospital of Guangdong Medical University; SBP, systolic blood pressure; CKD, chronic kidney disease; COPD, chronic obstructive pulmonary disease; HB haemoglobin; WC, waist circumference; TC, total cholesterol; HDL-C, high-density lipoprotein-cholesterol; LDL-C, low-density lipoprotein-cholesterol; TG, triglyceride; Cr, creatinine; CC, creatinine clearance; TBIL, total bilirubin; LVEF, left ventricular ejection fraction; AV-Vmax, maximum velocity of blood flow through the aortic valve; AV-MG, mean gradient across the aortic valve; AVA, aortic valve area; PAH, pulmonary arterial hypertension; ACEI, angiotensin converting enzyme inhibitor; ARB, angiotensin receptor blocker; CCB, calcium channel blocker.

**Supplementary Table 2.** Clinical characteristics of the participants, stratified according to only EUROSCORE II.

|  | Total(N=544) | Low Risk(n=316) | High Risk(n=228) | P value |
| --- | --- | --- | --- | --- |
| EUROSCORE | 0.03 (0.02-0.05) | 0.02 (0.01-0.03) | 0.06 (0.05-0.09) | <0.001 |
| **General characteristics** | | | | |
| Hospital (%) |  |  |  |  |
| A | 29(5.33) | 17(5.38) | 12(5.26) | 0.907 |
| B | 454(83.46) | 262(82.91) | 192(84.21) | |
| C | 61(11.21) | 37(11.71) | 24(10.53) | |
| Age (years) | 66.00 (57.00-74.00) | 65.50 (56.75-74.00) | 66.00 (58.00-75.00) | 0.089 |
| Gender(%) |  |  |  |  |
| Male | 300(55.15) | 197(62.34) | 103(45.18) | <0.001 |
| Female | 244(44.85) | 119(37.66) | 125(54.82) | |
| SBP (mmHg) | 126.00 (111.00-140.00) | 128.00 (115.00-143.00) | 122.00 (108.00-137.00) | 0.002 |
| Weight (kg) | 58.00 (50.00-65.00) | 60.00 (53.00-67.00) | 54.10 (48.00-62.78) | <0.001 |
| **State assessment** | | | | |
| Status at the first clinic visit (%) | | | | |
| elective | 518(54.5) | 316(100.00) | 202(88.60) | <0.001 |
| urgent | 26(4.78) | 0(0.00) | 26(11.40) | |
| The urgency of the surgery | | | | |
| 0 | 264(48.53) | 216(68.35) | 48(21.05) | <0.001 |
| non-CABG | 276(50.74) | 100(31.65) | 176(77.19) | |
| 2 | 3(0.55) | 0(0.00) | 3(1.31) |  |
| 3+ | 1(0.18) | 0(0.00) | 1(0.43) |  |
| NYHA ( first visit） | | | | |
| I | 129(23.71) | 102(32.28) | 27(11.84) | <0.001 |
| II | 176(32.35) | 123(38.92) | 53(23.25) | |
| III | 194(35.66) | 82(25.95) | 112(49.12) | |
| IV | 45(8.27) | 9(2.85) | 36(15.79) | |
| **Comorbidities** |  |  |  |  |
| Hypertension | 213(39.15) | 129(40.82) | 84(36.84) | 0.396 |
| Active endocarditis | 12(2.21) | 2(0.63) | 10(4.39) | 0.008 |
| Previous major cardiac surgery | 210(38.60) | 60(18.99) | 150(65.79) | <0.001 |
| Atrial fibrillation | 88(16.18) | 44(13.92) | 44(19.30) | 0.118 |
| Stroke | 36(6.62) | 24(7.59) | 12(5.26) | 0.366 |
| CKD | 33(6.07) | 15(4.75) | 18(7.89) | 0.182 |
| Extra-cardiac artery lesions | 33(6.07) | 13(4.11) | 20(8.77) | 0.039 |
| COPD | 22 (4.00) | 12 (3.80) | 10 (4.39) | 0.902 |
| Diabetes mellitus | 82 (15.07) | 51 (16.14) | 31 (13.60) | 0.486 |
| History of gout | 20 (3.68) | 11 (3.48) | 9 (3.95) | 0.957 |
| History of smoking | 149 (27.39) | 93 (29.43) | 56 (24.56) | 0.246 |
| History of drinking | 84 (15.44) | 51 (16.14) | 33 (14.47) | 0.682 |
| **Laboratory parameters** | | | | |
| HB (g/L) | 126.00 (113.00-139.00) | 128.50 (116.00-141.00) | 123.00 (108.00-136.00) | 0.001 |
| WBC (10^9^/L) | 6.96 (5.58-8.49) | 7.00 (5.60-8.40) | 6.92 (5.56-8.65) | 0.619 |
| PLT (10^9^/L) | 199.50 (160.00-243.00) | 203.00 (165.50-242.50) | 193.00 (148.75-243.00) | 0.100 |
| TC (mmol/L) | 4.53 (3.80-5.10) | 4.53 (3.90-5.33) | 4.53 (3.64-4.70) | 0.002 |
| HDL-C (mmol/L) | 1.14 (0.95-1.28) | 1.14 (0.95-1.31) | 1.14 (0.95-1.22) | 0.334 |
| LDL-C (mmol/L) | 2.87 (2.34-3.26) | 2.87 (2.40-3.44) | 2.86 (2.23-2.90) | 0.005 |
| TG (mmol/L) | 1.23 (0.86-1.40) | 1.27 (0.87-1.50) | 1.13 (0.82-1.27) | 0.02 |
| eGFR (mL/min/1.73m^2^) | 59.30 (44.53-77.61) | 64.19 (49.89-84.44) | 50.45 (38.83-69.91) | <0.001 |
| UREA (mmol/L) | 6.30 (4.90-8.00) | 6.20 (4.80-7.33) | 6.57 (5.00-8.90) | 0.001 |
| TBIL (μmol/L) | 12.80 (9.70-18.20) | 11.95 (9.38-15.90) | 13.80 (10.50-20.27) | 0.001 |
| Blood glucose (mmol/L) | 5.00 (4.40-5.80) | 4.90 (4.30-5.70) | 5.00 (4.50-6.03) | 0.048 |
| Uric acid (mmol/L) | 411.00 (325.00-494.15) | 402.90 (318.00-476.00) | 424.38 (337.50-524.25) | 0.029 |
| Elevated NT-proBNP (%) | 275(50.55) | 155(49.05) | 120(52.63) | 0.461 |
| Troponin＞40 (%) | 15(2.76) | 8(2.53) | 7(3.07) | 0.910 |
| Echocardiogram |  |  |  |  |
| LVEF (%) | 65.00 (56.00-72.00) | 66.00 (58.00-73.00) | 62.00 (49.75-69.25) | <0.001 |
| Aortic valve bilobate malformation (%) | 83(15.26) | 53(16.77) | 30(13.16) | 0.300 |
| AV-Vmax (m/s) | 4.20 (3.72-4.79) | 4.15 (3.63-4.73) | 4.30 (3.80-4.80) | 0.047 |
| AV-MG (mmHg) | 42.50 (33.00-55.25) | 41.00 (32.00-54.00) | 44.50 (35.00-57.00) | 0.018 |
| AVA (cm^2^) | 0.84 (0.70-1.00) | 0.90 (0.70-1.00) | 0.80 (0.63-0.98) | <0.001 |
| PAH (%) |  |  |  |  |
| 0-30mmHg | 233(42.83) | 150(47.47) | 73(32.02) | <0.001 |
| 31-55mmHg | 258(47.43) | 140(44.30) | 118(51.75) | |
| >55mmHg | 63(11.58) | 26(8.23) | 37(16.23) | |
| Aortic regurgitation (%) | | | | |
| Non | 37(6.80) | 25(7.91) | 12(5.26) | 0.005 |
| Mild | 256(47.06) | 149(47.15) | 107(46.93) | |
| Moderate | 171(31.43) | 109(34.49) | 62(27.19) | |
| Severe | 80(14.71) | 33(10.44) | 47(20.61) | |
| Aortic stenosis (%) |  |  |  |  |
| Moderate | 230(42.28) | 152(48.10) | 78(34.21) | <0.001 |
| Severe | 314(57.72) | 164(51.90) | 150(65.79) | |
| Mitral regurgitation (%) | | | | |
| Non | 120(22.06) | 83(26.27) | 37(16.23) | <0.001 |
| Mild | 279(51.29) | 166(52.53) | 113(49.56) | |
| Moderate | 106(19.49) | 48(15.19) | 58(25.44) | |
| Severe | 39(7.17) | 19(6.01) | 20(8.77) |  |
| Mitral stenosis (%) | | | | |
| Non | 402(73.90) | 245(77.53) | 157(68.86) | 0.030 |
| Mild | 51(9.38) | 31(9.81) | 20(8.77) |  |
| Moderate | 46(8.46) | 20(6.33) | 26(11.40) | |
| Severe | 45(8.27) | 20(6.33) | 25(10.96) | |
| **Cardiovascular medical therapy** | | | | |
| Anti-plate medicine (%) | 206(37.87) | 141(44.62) | 65(28.51) | <0.001 |
| Stains (%) | 200(36.76) | 128(40.51) | 72(31.58) | 0.041 |
| ACEI/ARB (%) | 142(26.10) | 95(30.06) | 47(20.61) | 0.017 |
| β-receptor blockers (%) | 286(52.57) | 164(51.90) | 122(53.51) | 0.776 |
| CCB (%) | 81(14.89) | 57(18.04) | 24(10.53) | 0.021 |
| Diuretic drug (%) | 384(70.59) | 190(60.13) | 194(85.09) | <0.001 |
| Insulin (%) | 47(8.64) | 20(6.33) | 27(11.84) | 0.021 |
| Oral antidiabetic drug (%) | 73(13.42) | 43(13.61) | 30(13.16) | 0.981 |
| Oral anticoagulants (%) | 257(47.24) | 105(33.23) | 152(66.67) | <0.001 |

Continuous variables: Median(IQR) or Mean ± Standard Deviation; categorical variables: N (%). *P* ≤ 0.05 was considered statistically significant. *P* >0.05, no significant.

A, Second Affiliated Hospital of Shantou University Medical College, B, the First Affiliated Hospital of Sun Yat-sen University, C, the Affiliated Hospital of Guangdong Medical University; SBP, systolic blood pressure; CKD, chronic kidney disease; COPD, chronic obstructive pulmonary disease; HB haemoglobin; WC, waist circumference; TC, total cholesterol; HDL-C, high-density lipoprotein-cholesterol; LDL-C, low-density lipoprotein-cholesterol; TG, triglyceride; Cr, creatinine; CC, creatinine clearance; TBIL, total bilirubin; LVEF, left ventricular ejection fraction; AV-Vmax, maximum velocity of blood flow through the aortic valve; AV-MG, mean gradient across the aortic valve; AVA, aortic valve area; PAH, pulmonary arterial hypertension; ACEI, angiotensin converting enzyme inhibitor; ARB, angiotensin receptor blocker; CCB, calcium channel blocker.

**Supplementary Table 3.** Echocardiographic characteristics and medical therapy of the participants, stratified according to both EUROSCORE II and surgical status.

|  | total  (N=544) | Low risk  with AVR  (n=154) | Low risk without AVR  (n=162) | High risk  with AVR  (n=172) | High risk without AVR  (n=56) | P value |
| --- | --- | --- | --- | --- | --- | --- |
| **Echocardiogram** | | | | | | |
| LVEF (%) | 65.00 (56.00-72.00) | 66.00 (58.00-73.00) | 66.00 (59.25-72.00) | 63.00 (51.75-70.00) | 61.00 (47.50-66.50) | <0.001 |
| Aortic valve bilobate malformation (%) | 83 (15.26) | 29 (18.83) | 24 (14.81) | 25 (14.53) | 5 (8.93) | 0.341 |
| AV-Vmax (m/s) | 4.20 (3.72-4.79) | 4.40 (4.00-5.00) | 3.94 (3.41-4.40) | 4.39 (3.96-4.84) | 4.05 (3.54-4.53) | <0.001 |
| AV-MG (mmHg) | 42.50 (33.00-55.25) | 46.50 (37.25-60.00) | 36.00 (26.25-45.75) | 46.00 (37.75-58.25) | 40.00 (28.00-49.50) | <0.001 |
| AVA (cm^2^) | 0.84 (0.70-1.00) | 0.86 (0.70-1.00) | 0.90 (0.72-1.10) | 0.80 (0.63-0.90) | 0.84 (0.64-1.02) | <0.001 |
| PAH (%) |  |  |  |  |  |  |
| 0-30mmHg | 223 (40.99) | 80 (51.95) | 70 (43.21) | 62 (36.05) | 11 (19.64) | <0.001 |
| 31-55mmHg | 258 (47.43) | 61 (39.61) | 79 (48.77) | 87 (50.58) | 31 (55.36) |  |
| >55mmHg | 63 (11.58) | 13 (8.44) | 13 (8.02) | 23 (13.37) | 14 (25.00) |  |
| Aortic regurgitation (%) | | | | | |  |
| Non | 37 (6.80) | 12 (7.79) | 13 (8.02) | 9 (5.23) | 3 (5.36) | 0.001 |
| Mild | 256 (47.06) | 67 (43.51) | 82 (50.62) | 70 (40.70) | 37 (66.07) |  |
| Moderate | 171 (31.43) | 58 (37.66) | 51 (31.48) | 53 (30.81) | 9 (16.07) |  |
| Severe | 80 (14.71) | 17 (11.04) | 16 (9.88) | 40 (23.26) | 7 (12.50) |  |
| Aortic stenosis (%) | | | | | |  |
| Moderate | 230 (42.28) | 53 (34.42) | 99 (61.11) | 50 (29.07) | 28 (50.00) | <0.001 |
| Severe | 314 (57.72) | 101 (65.58) | 63 (38.89) | 122 (70.93) | 28 (50.00) |  |
| Mitral regurgitation (%) | | | | | |  |
| Non | 120 (22.06) | 42 (27.27) | 41 (25.31) | 24 (13.95) | 13 (23.21) | 0.016 |
| Mild | 279 (51.29) | 85 (55.19) | 81 (50.00) | 86 (50.00) | 27 (48.21) |  |
| Moderate | 106 (19.49) | 18 (11.69) | 30 (18.52) | 45 (26.16) | 13 (23.21) |  |
| Severe | 39 (7.17) | 9 (5.84) | 10 (6.17) | 17 (9.88) | 3 (5.36) |  |
| Mitral stenosis (%) | | | | | |  |
| Non | 402 (73.90) | 122 (79.22) | 123 (75.93) | 113 (65.70) | 44 (78.57) | 0.063 |
| Mild | 51 (9.38) | 12 (7.79) | 19 (11.73) | 15 (8.72) | 5 (8.93) |  |
| Moderate | 46 (8.46) | 9 (5.84) | 11 (6.79) | 23 (13.37) | 3 (5.36) |  |
| Severe | 45 (8.27) | 11 (7.14) | 9 (5.56) | 21 (12.21) | 4 (7.14) |  |
| **Cardiovascular medical therapy** | | | | | | |
| Anti-plate medicine (%) | 206 (37.87) | 63 (40.91) | 78 (48.15) | 42 (24.42) | 23 (41.07) | <0.001 |
| Stains (%) | 200 (36.76) | 46 (29.87) | 82 (50.62) | 38 (22.09) | 34 (60.71) | <0.001 |
| ACEI/ARB (%) | 142 (26.10) | 27 (17.53) | 68 (41.98) | 24 (13.95) | 23 (41.07) | <0.001 |
| β-receptor blockers (%) | 286 (52.57) | 85 (55.19) | 79 (48.77) | 94 (54.65) | 28 (50.00) | 0.612 |
| CCB (%) | 81 (14.89) | 17 (11.04) | 40 (24.69) | 16 (9.30) | 8 (14.29) | <0.001 |
| Diuretic drug (%) | 384 (70.59) | 117 (75.97) | 73 (45.06) | 157 (91.28) | 37 (66.07) | <0.001 |
| Insulin (%) | 47 (8.64) | 6 (3.90) | 14 (8.64) | 17 (9.88) | 10 (17.86) | 0.013 |
| Oral antidiabetic drug (%) | 73 (13.42) | 20 (12.99) | 23 (14.20) | 21 (12.21) | 9 (16.07) | 0.881 |
| Oral anticoagulants (%) | 88 (87.14) | 17 (10.49) | 139 (10.81) | 13 (13.21) | 257 (27.24) | <0.001 |

Continuous datasets were tested for normality using the Shapiro–Wilk method, and the data are presented as as mean ±SD for normal distribution, or median and interquartile range for skewed data. Categorical data are presented as counts and percentages (%). Homogeneous datasets compared using ANOVA, heterogeneous using Kruskal–Wallis test. Student’s t-test or ANOVA was used to evaluate differences between groups with respect to continuous data, and Pearson’s chi-square test or Fisher’s exact test was used to compare categorical datasets, as appropriate.

Group 1 (low risk (EuroSCORE II index <4%) with AVR); Group 2 (low risk (EuroSCORE II index <4%) without AVR); Group 3 (high risk (EuroSCORE II index ≥4%) with AVR); Group 4 (high risk (EuroSCORE II index ≥4%) without AVR). *P* ≤ 0.05 was considered statistically significant. *P* >0.05, no significant.

LVEF, left ventricular ejection fraction, AV-Vmax, maximum flow velocity through the aortic valve, AV-MG, mean gradient across the aortic valve; AVA, aortic valve area; PAH, pulmonary arterial hypertension; ACEI, angiotensin converting enzyme inhibitor: ARB, angiotensin receptor blocker: CCB, calcium channel blocker.

**Supplementary Table 4** Results of the univariate and multivariate Cox analyses of the associations with EUROSCORE II and AVR status.

|  | Univariate analysis | P value | multivariate analysis | P value |
| --- | --- | --- | --- | --- |
|  | HR (95% CI) |  | HR (95% CI) |  |
| Age | 1.042(1.025-1.058) | <0.001 |  |  |
| Gender | 0.956(0.664-1.376) | 0.808 |  |  |
| SBP | 1.008(1.000-1.016) | 0.052 | 0.997(0.987-1.007) | 0.545 |
| Weight | 0.984(0.967-1.002) | 0.083 | 0.980(0.960-1.001) | 0.067 |
| Smoking | 1.121(0.752-1.671) | 0.576 |  |  |
| Drinking | 0.902(0.539-1.509) | 0.694 |  |  |
| Coronary disease | 1.483(0.984-2.238) | 0.06 |  |  |
| Myocardial infarction | 2.360(1.150-4.843) | 0.019 |  |  |
| Previous major cardiac surgery | 0.280(0.177-0.444) | <0.001 |  |  |
| Stroke | 2.728(1.607-4.629) | <0.001 | 1.539(0.821-2.886) | 0.179 |
| CKD | 3.183(1.873-5.408) | <0.001 | 1.583(0.841-2.981) | 0.155 |
| Extra-cardiac artery lesions | 1.924(0.894-4.139) | 0.094 | 0.669(0.296-1.513) | 0.335 |
| COPD | 1.710(0.833-3.510) | 0.144 |  |  |
| Diabetes mellitus | 2.052(1.340-3.144) | 0.001 | 1.382(0.832-2.298) | 0.212 |
| Atrial fibrillation | 1.802(1.189-2.731) | 0.006 | 1.472(0.910-2.380) | 0.115 |
| Hypertension | 1.496(1.040-2.152) | 0.03 | 0.856(0.529-1.385) | 0.527 |
| Stroke | 4.210(2.254-7.863) | <0.001 | 2.334(1.158-4.703) | 0.018 |
| NYHA of first visit |  | 0.189 |  |  |
| II | 0.700(0.417-1.175) | 0.177 |  |  |
| III | 1.082(0.675-1.737) | 0.742 |  |  |
| IV | 1.290(0.640-2.600) | 0.477 |  |  |
| Extracardiac artery lesions | 1.104(0.538-2.263) | 0.788 |  |  |
| WBC | 1.055(1.017-1.094) | 0.004 | 1.056(1.013-1.100) | 0.010 |
| HB | 0.988(0.981-0.995) | 0.001 |  |  |
| PLT | 0.998(0.996-1.001) | 0.132 |  |  |
| TC | 0.999(0.853-1.170) | 0.99 |  |  |
| HDLC | 0.705(0.371-1.340) | 0.286 |  |  |
| LDLC | 1.038(0.849-1.270) | 0.715 |  |  |
| TG | 0.702(0.488-1.010) | 0.056 | 0.888(0.603-1.308) | 0.547 |
| TBIL | 1.012(1.006-1.017) | <0.001 | 1.013(1.006-1.020) | <0.001 |
| Blood glucose | 1.058(1.017-1.100) | 0.005 | 1.022(0.964-1.085) | 0.463 |
| Uric acid | 1.002(1.001-1.003) | 0.005 |  |  |
| Elevated NT-proBNP | 2.177(1.473-3.217) | <0.001 | 1.314(0.851-2.029) | 0.218 |
| Troponin＞40 | 1.139(0.279-4.653) | 0.856 |  |  |
| Albumin | 0.915(0.885-0.946) | <0.001 | 0.961(0.917-1.007) | 0.097 |
| Status at the first clinic visit | 15.992(9.656-26.486) | <0.001 |  |  |
| The urgency of the surgery | 0.323(0.216-0.483) | <0.001 |  |  |
| LVEF | 0.983(0.969-0.996) | 0.010 |  |  |
| Aortic valve bilobate malformation | 0.554(0.298-1.032) | 0.063 | 0.608(0.318-1.164) | 0.133 |
| AV-Vmax | 0.799(0.632-1.009) | 0.060 | 0.982(0.818-1.179) | 0.843 |
| AV-MG | 0.991(0.980-1.001) | 0.079 | 1.002(0.988-1.015) | 0.824 |
| AVA | 0.909(0.476-1.738) | 0.774 |  |  |
| PAH | 1.538(1.173-2.017) | 0.002 |  |  |
| Aortic regurgitation | 0.960(0.772-1.194) | 0.715 |  |  |
| Aortic stenosis | 0.794(0.552-1.143) | 0.214 |  |  |
| Mitral regurgitation | 1.096(0.882-1.361) | 0.409 |  |  |
| Mitral stenosis | 1.042(0.870-1.248) | 0.652 |  |  |
| Anti-plate medicine | 1.267(0.867-1.851) | 0.221 |  |  |
| Stains | 1.788(1.237-2.583) | 0.002 | 0.844(0.537-1.326) | 0.462 |
| ACEI/ARB | 1.873(1.287-2.725) | 0.001 | 1.374(0.878-2.150) | 0.164 |
| β-receptor blockers | 1.149(0.798-1.655) | 0.454 |  |  |
| CCB | 1.464(0.934-2.295) | 0.096 | 1.171(0.692-1.982) | 0.555 |
| Diuretic drug | 0.791(0.536-1.165) | 0.235 |  |  |
| Insulin | 2.430(1.484-3.979) | <0.001 |  |  |
| Oral antidiabetic drug | 1.045(0.597-1.829) | 0.878 |  |  |
| Oral anticoagulants | 0.383(0.258-0.567) | <0.001 | 0.807(0.470-1.385) | 0.436 |
| Group |  | <0.001 |  | <0.001 |
| 2 | 5.966(3.033-11.737) | <0.001 | 3.705(1.738-7.896) | 0.001 |
| 3 | 1.972(0.938-4.144) | 0.073 | 1.368(0.627-2.984) | 0.431 |
| 4 | 12.168(5.958-24.848) | <0.001 | 6.891(3.083-15.401) | <0.001 |

The univariate and multivariate Cox regression analyses of the EUROSCORE II with the all-cause mortality of the 544 participants.

*P* ≤ 0.05 was considered statistically significant. *P* >0.05, no significant.

A, Second Affiliated Hospital of Shantou University Medical College, B, the First Affiliated Hospital of Sun Yat-sen University, C, the Affiliated Hospital of Guangdong Medical University; SBP, systolic blood pressure; CKD, chronic kidney disease; COPD, chronic obstructive pulmonary disease; HB haemoglobin; WC, waist circumference; TC, total cholesterol; HDL-C, high-density lipoprotein-cholesterol; LDL-C, low-density lipoprotein-cholesterol; TG, triglyceride; Cr, creatinine; CC, creatinine clearance; TBIL, total bilirubin; LVEF, left ventricular ejection fraction; AV-Vmax, maximum velocity of blood flow through the aortic valve; AV-MG, mean gradient across the aortic valve; AVA, aortic valve area; PAH, pulmonary arterial hypertension; ACEI, angiotensin converting enzyme inhibitor; ARB, angiotensin receptor blocker; CCB, calcium channel blocker.

**Supplementary Table 5** The comparison of SAVR and TAVR on the all-cause mortality of the participants.

| variables | SAVR (n=331) | TAVR (n=164) | P value |
| --- | --- | --- | --- |
| Model 1 | Reference | 1.07 (0.40-2.87) | 0.886 |
| Model 2 | Reference | 0.73 (0.22-2.41) | 0.608 |
| Model 3 | Reference | 0.46 (0.12-1.70) | 0.242 |
| Model 4 | Reference | 0.84 (0.15-4.80) | 0.848 |

Model 1 was adjusted for smoking status, alcohol consumption status, diabetes, atrial fibrillation, hypertension, gout, systolic blood pressure, and body mass;

Model 2 was adjusted for the parameters in model 1, with the addition of WBC, RBC, PLT, LDLC, TG, TBIL, NT-proBNP, troponin, and albumin;

Model 3 was adjusted for the parameters in model 2, plus aortic valve deformity, rheumatic heart disease, AV-Vmax, AV-MG, the degree of AVA mitral insufficiency, and mitral stenosis;

Model 4 was adjusted for the parameters in Model 3, with the addition of anti-platelet agent, statin, ACEI, ARB, β-blocker, CCB, diuretic, insulin, oral antidiabetic drug, and oral anticoagulant use.

*P* ≤ 0.05 was considered statistically significant. *P* >0.05, no significant.

**Supplementary Table 6.** The effect of whether AVR (SAVR or TAVI) on low risk participants(EUROSCORE II <4%).

| variables | low risk without surgery  (n=162) | low risk with surgery  (n=154) | P value |
| --- | --- | --- | --- |
| Model 1 | Reference | 0.137 (0.071-0.264) | **<0.001** |
| Model 2 | Reference | 0.128 (0.062-0.267) | **<0.001** |
| Model 3 | Reference | 0.134 (0.060-0.297) | **<0.001** |
| Model 4 | Reference | 0.230 (0.118-0.857) | **<0.001** |

Model 1 was adjusted for smoking status, alcohol consumption status, diabetes, atrial fibrillation, hypertension, gout, systolic blood pressure, and body mass;

Model 2 was adjusted for the parameters in model 1, with the addition of WBC, RBC, PLT, LDLC, TG, TBIL, NTproBNP, troponin, and albumin;

Model 3 was adjusted for the parameters in model 2, plus aortic valve deformity, rheumatic heart disease, AV-Vmax, AV-MG, the degree of AVA mitral insufficiency, and mitral stenosis;

Model 4 was adjusted for the parameters in Model 3, with the addition of anti-platelet agent, statin, ACEI, ARB, β-blocker, CCB, diuretic, insulin, oral antidiabetic drug, and oral anticoagulant use.

*P* ≤ 0.05 was considered statistically significant. *P* >0.05, no significant.

**Supplementary Table 7.** Results of the Cox multivariate analysis of all-cause mortality, using the calculated optimal cut-off value of EUROSCORE II (2.23%), for the participants who had not undergone AVR

| Variables | HR (95% CI) | P value |
| --- | --- | --- |
| Age | 1.011 ( (0.982-1.041) | 0.456 |
| Gender | 0.623 (0.334-1.161) | 0.136 |
| Smoke | 1.177 (0.652-2.128) | 0.589 |
| Diabetes | 1.835 (0.818-4.117) | 0.141 |
| Previous major cardiac surgery | 0.591 (0.119-2.941) | 0.521 |
| Hypertension | 0.880 (0.493-1.571) | 0.666 |
| L-DLC | 0.823 (0.613-1.105) | 0.196 |
| Cr | 1.002 (1.001-1.003) | 0.008 |
| LVEF | 0.943 (0.901-0.986) | 0.010 |
| AV-Vmax | 0.560 (0.068-4.581) | 0.588 |
| AV-MG | 0.971 (0.907-1.039) | 0.394 |
| AVA | 0.944 (0.293-3.034) | 0.922 |
| Aortic regurgitation | 1.604 (1.11-2.317) | 0.012 |
| Mitral regurgitation | 1.015 (0.713-1.444) | 0.935 |
| Mitral stenosis | 1.431 (1.054-1.942) | 0.022 |
| Anti-plate medicine | 0.849 (0.463-1.557) | 0.597 |
| ACEI/ARB | 1.550 (0.922-2.606) | 0.098 |
| Stains | 0.831 (0.458-1.507) | 0.542 |
| Insulin | 1.564 (0.686-3.563) | 0.287 |
| Oral anti-diabetic drug | 0.713 (0.297-1.71) | 0.448 |
| **EUROSCORE II** (cut-off point 2.23%) | 2.111 (1.069-4.166) | 0.031 |

The higher EUROSCORE II (≥2.23%) group had a 2.111-fold higher risk of all-cause mortality (HR 2.111, 95% CI (1.069–4.166), *P*=0.031), according to Cox multivariate analysis.

LDL-C, low-density lipoprotein-cholesterol; TG, triglyceride; Cr, creatinine; CC, creatinine clearance; TBIL, total bilirubin; LVEF, left ventricular ejection fraction; AV-Vmax, maximum velocity of blood flow through the aortic valve; AV-MG, mean gradient across the aortic valve; AVA, aortic valve area; ACEI, angiotensin converting enzyme inhibitor; ARB, angiotensin receptor blocker.


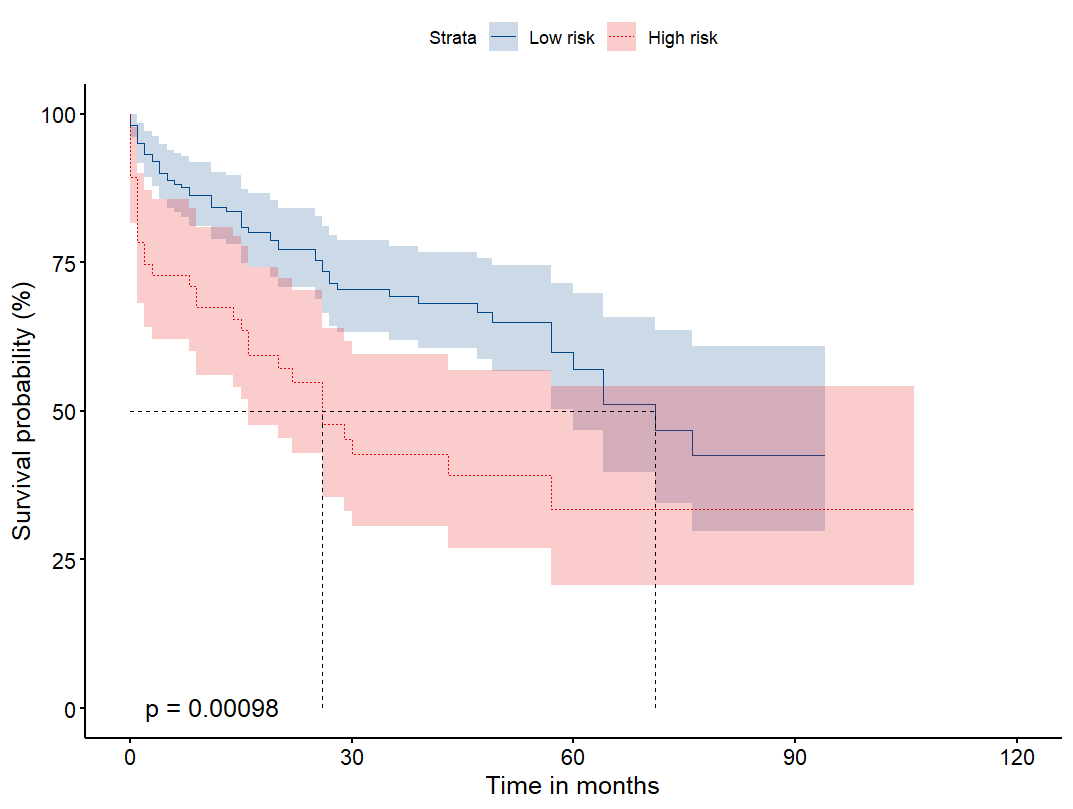


**Supplementary Fig. 1**. Kaplan–Meier survival curves for the participants who had not undergone AVR, categorized according to EUROSCORE II

The low-risk group of participants (EUROSCORE II <4%) showed more cumulative survival time than high-risk group between those who had not undergone AVR(*P*=0.00098).

*P* ≤ 0.05 was considered statistically significant. *P* >0.05, no significant.


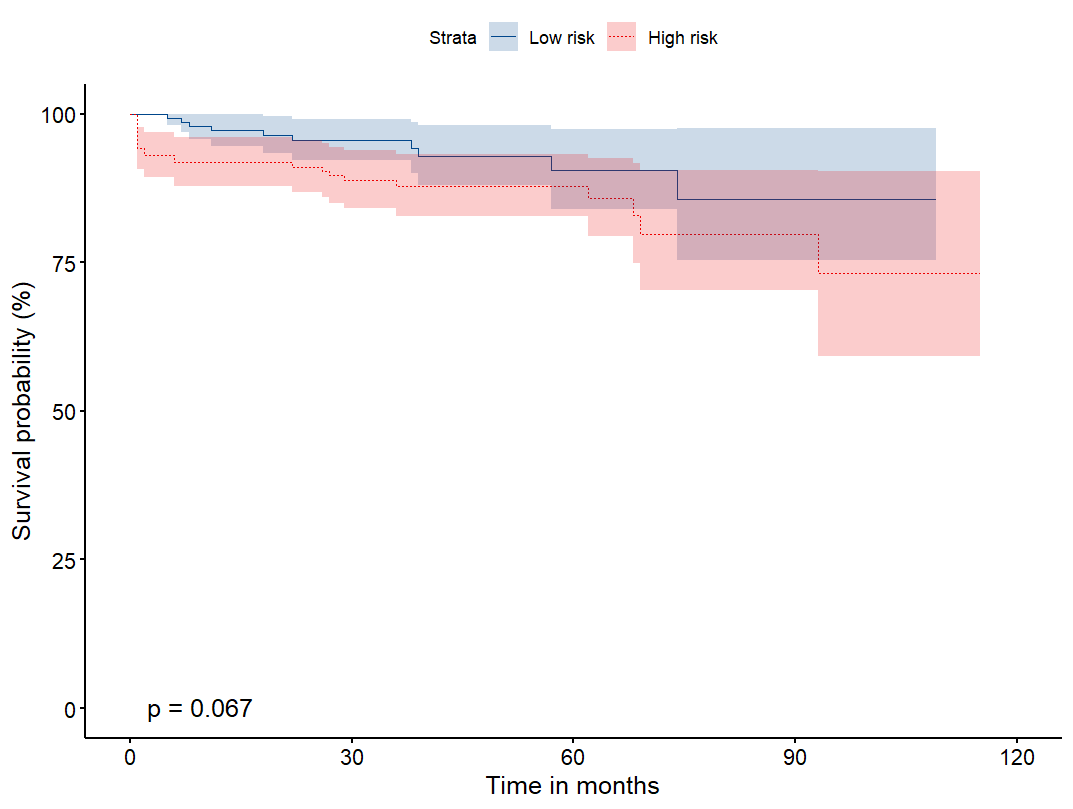


**Supplementary Fig. 2.** Kaplan–Meier survival curves for the participants who underwent AVR, categorized according to EUROSCORE II.

In patients with AVR, the low-risk group of participants (EUROSCORE II <4%) showed no difference in participants with high-risk group (EUROSCORE II≥4%) (*P*=0.067).

*P* ≤ 0.05 was considered statistically significant. *P* >0.05, no significant.


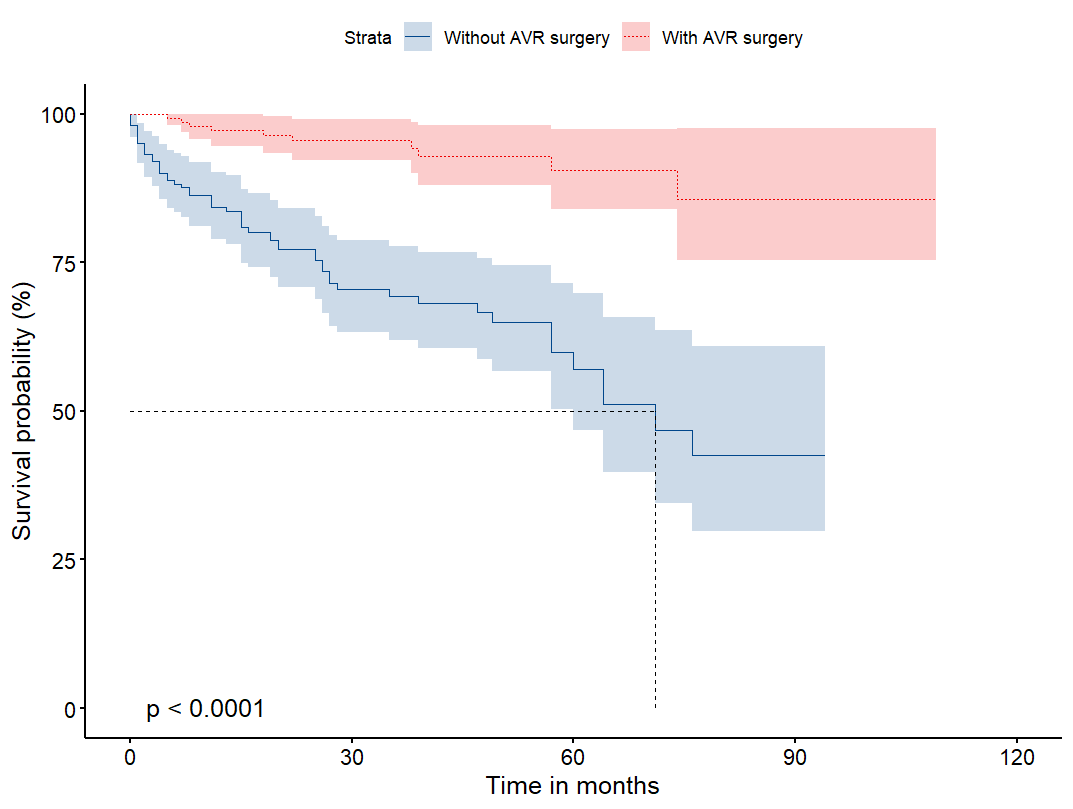


**Supplementary Fig. 3.** Kaplan–Meier survival curves for the participants in the low-risk group, according to whether or not they had undergone AVR

*P* ≤ 0.05 was considered statistically significant. *P* >0.05, no significant.


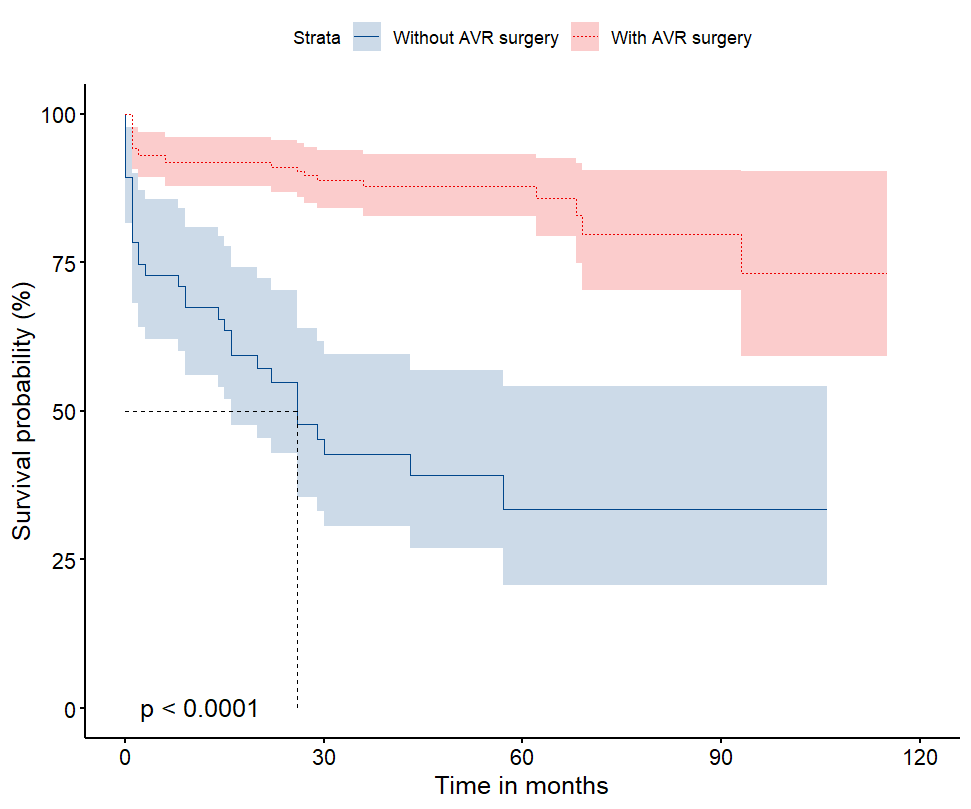


**Supplementary Fig. 4.** Kaplan–Meier survival curves for the participants in the high-risk group, according to whether or not they had undergone AVR

0: without AVR(SAVR or TAVI); 1: with AVR(SAVR or TAVI)

*P* ≤ 0.05 was considered statistically significant. *P* >0.05, no significant.


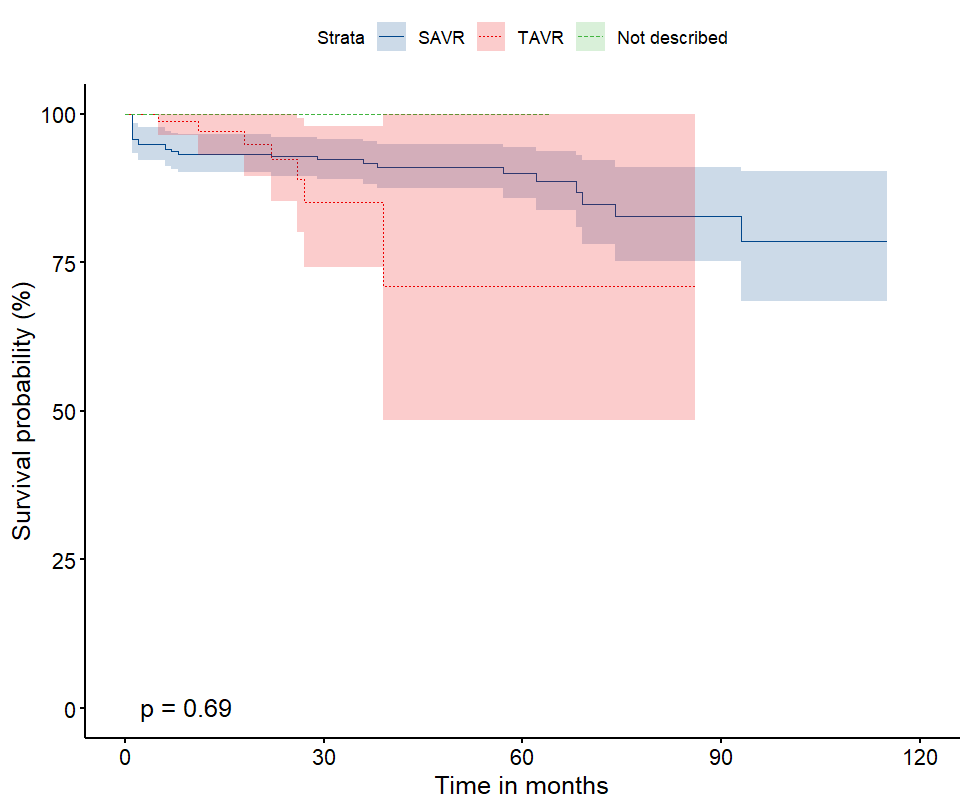


**Supplementary Fig. 5.** Kaplan–Meier survival curves for the participants who underwent SAVR or TAVI

There was no difference between those who underwent SAVR or TAVI (*P*=0.69).

*P* ≤ 0.05 was considered statistically significant. *P* >0.05, no significant.


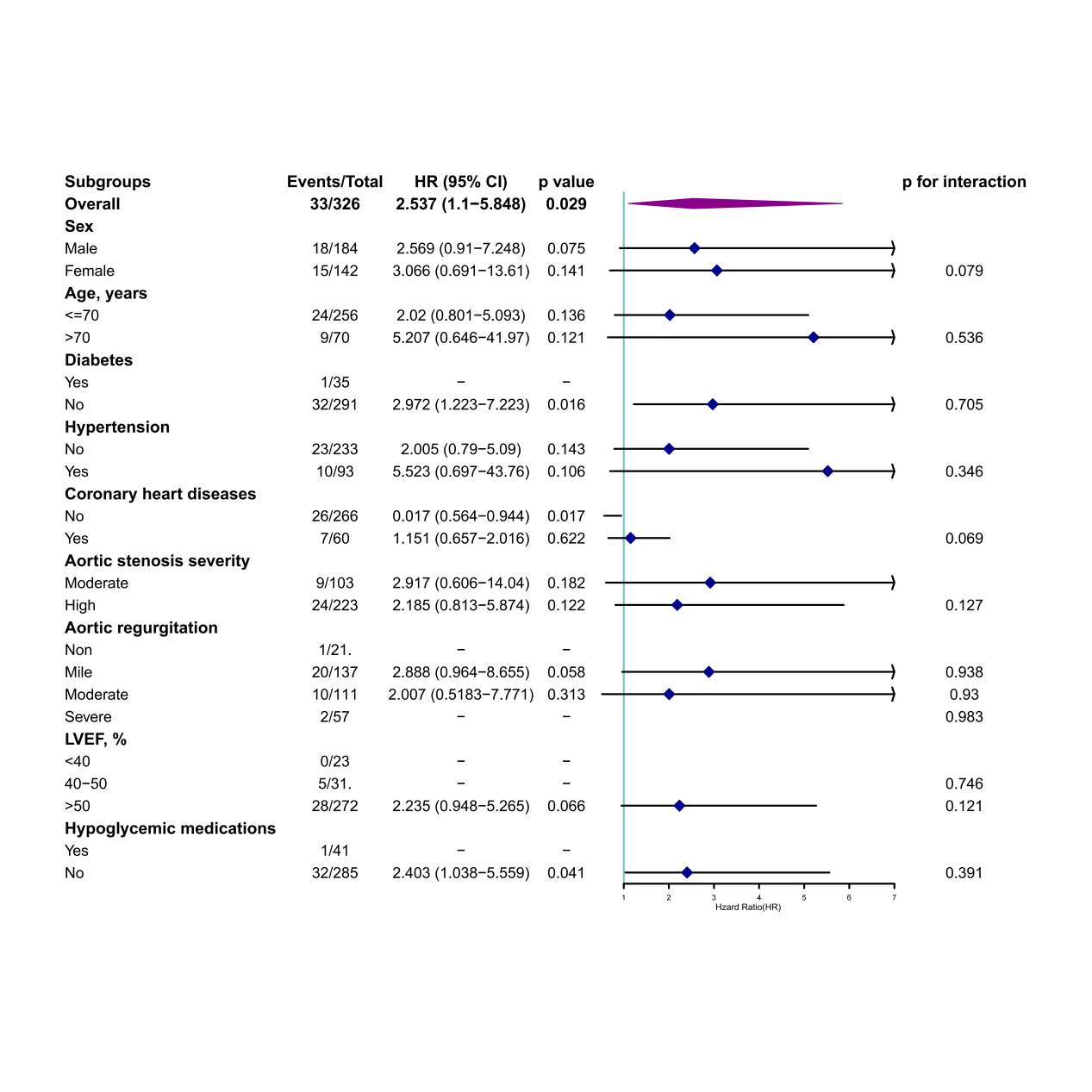


**Supplementary Fig. 6.** Results of the subgroup analysis of the association between EUROSCORE II and AVR status and incident all-cause mortality.

Subgroup analyses were performed after the stratification of the participants according to baseline sex, age (≤ 70 or >70 years-old), BMI (<24 or ≥24 kg/m^2^), diabetes, hypertension, CHD, and the severity of AS (moderate or severe), to assess the consistency of the prognostic use of EUROSCORE II for all-cause mortality.

HR, hazard ratio; CI, confidence interval.


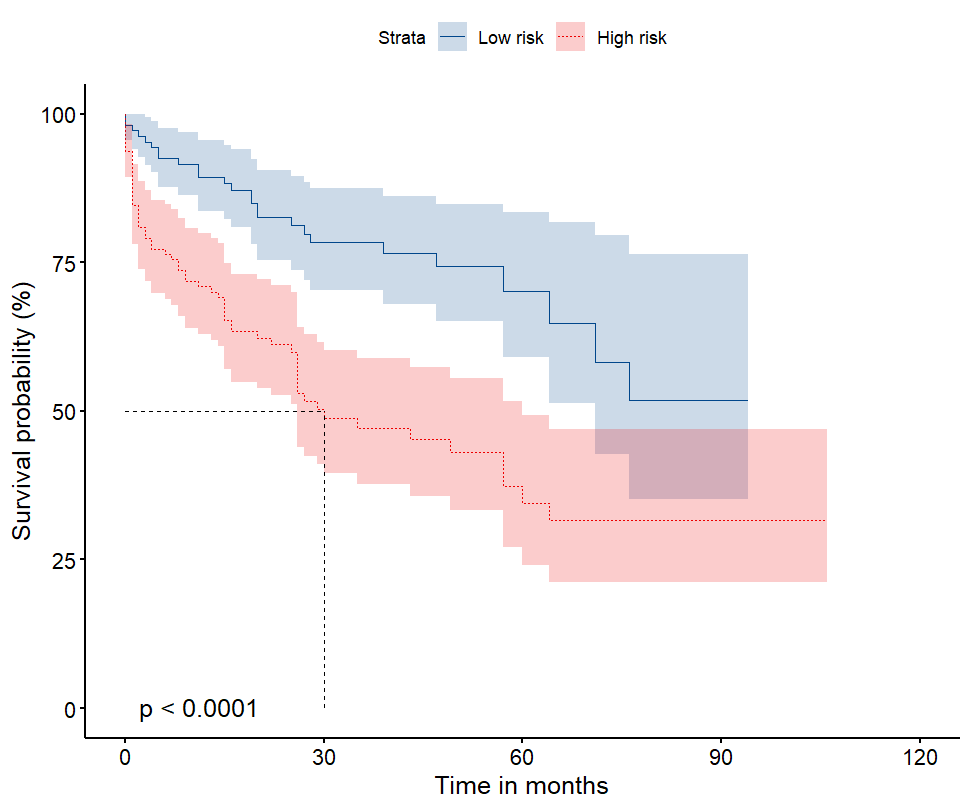


**Supplementary Fig. 7.** Kaplan–Meier curves for all-cause mortality, using the identified optimal cut-off value of EUROSCORE II (2.23%), for the participants who had not undergone AVR. Kaplan–Meier analysis demonstrated that participants with a EUROSCORE II >2.23% had a higher risk of all-cause mortality than those with values <2.23% (log-rank, *P*<0.001).
